# Crosstalk between Long Non Coding RNAs, microRNAs and DNA Damage Repair in Prostate Cancer: New Therapeutic Opportunities?

**DOI:** 10.3390/cancers14030755

**Published:** 2022-01-31

**Authors:** Folake Orafidiya, Lin Deng, Charlotte Lynne Bevan, Claire Emily Fletcher

**Affiliations:** Imperial Centre for Translational and Experimental Medicine, Imperial College London, Hammersmith Hospital, Ducane Road, London W12 0NN, UK; f.orafidiya@imperial.ac.uk (F.O.); denglin1986@gmail.com (L.D.); charlotte.bevan@imperial.ac.uk (C.L.B.)

**Keywords:** DNA damage response, non-coding RNA, microRNA, long non-coding RNA, DNA damage, DNA repair, prostate cancer

## Abstract

**Simple Summary:**

Non-coding RNAs are a type of genetic material that doesn’t make protein, but performs diverse regulatory functions. In prostate cancer, most treatments target proteins, and resistance to such therapies is common, leading to disease progression. Targeting non-coding RNAs may provide alterative treatment options and potentially overcome drug resistance. Major types of non-coding RNAs include tiny ‘microRNAs’ and much longer ‘long non-coding RNAs’. Scientific studies have shown that these form a major part of the human genome, and play key roles in altering gene activity and determining the fate of cells. Importantly, in cancer, their activity is altered. Recent evidence suggests that microRNAs and long non-coding RNAs play important roles in controlling response to DNA damage. In this review, we explore how different types of non-coding RNA interact to control cell DNA damage responses, and how this knowledge may be used to design better prostate cancer treatments and tests.

**Abstract:**

It is increasingly appreciated that transcripts derived from non-coding parts of the human genome, such as long non-coding RNAs (lncRNAs) and microRNAs (miRNAs), are key regulators of biological processes both in normal physiology and disease. Their dysregulation during tumourigenesis has attracted significant interest in their exploitation as novel cancer therapeutics. Prostate cancer (PCa), as one of the most diagnosed malignancies and a leading cause of cancer-related death in men, continues to pose a major public health problem. In particular, survival of men with metastatic disease is very poor. Defects in DNA damage response (DDR) pathways culminate in genomic instability in PCa, which is associated with aggressive disease and poor patient outcome. Treatment options for metastatic PCa remain limited. Thus, researchers are increasingly targeting ncRNAs and DDR pathways to develop new biomarkers and therapeutics for PCa. Increasing evidence points to a widespread and biologically-relevant regulatory network of interactions between lncRNAs and miRNAs, with implications for major biological and pathological processes. This review summarises the current state of knowledge surrounding the roles of the lncRNA:miRNA interactions in PCa DDR, and their emerging potential as predictive and diagnostic biomarkers. We also discuss their therapeutic promise for the clinical management of PCa.

## 1. Introduction

### 1.1. Diagnosis and Treatment of Prostate Cancer

Prostate cancer (PCa) is the most prevalent male cancer in the Western world and the second most frequent malignancy in men worldwide [1,2]. An array of treatment options including prostatectomy, radiotherapy (RT), ablative therapies and chemotherapy, alongside active surveillance for clinically-insignificant disease, contribute to favourable prognosis of early-stage, organ-confined PCa [3]. The androgen receptor (AR) is a key driver of PCa development and progression. Consequently, androgen deprivation therapy (ADT), which blocks androgen synthesis and/or inhibits AR action, remains the mainstay treatment of localised or locally-advanced intermediate/high-risk and recurrent disease. This has also been applied in the adjuvant/neo-adjuvant setting. However, resistance to ADT occurs almost inevitably, leading to lethal castration-resistant PCa (CRPC), in which AR continues to drive tumour growth in the absence of circulating androgens. In addition, patient presentation with de novo ADT-resistance or de novo metastatic disease can be observed [4]. Treatment options in all cases are limited, but second-generation anti-androgens/androgen-synthesis inhibitors such as enzalutamide and abiraterone respectively, have been shown to increase survival [5,6,7]. Their efficacy has resulted in their approval for use at earlier disease stages: abiraterone plus prednisone in non-metastatic CRPC [8], and enzalutamide for low-volume disease or prior to docetaxel treatment in metastatic hormone-sensitive PCa [9]. Most of the clinically approved interventions for PCa are designed around protein coding constituents of the human genome, and these treatments inevitably culminate in resistance. Hence, there is a need to explore novel pathways in disease management.

### 1.2. The Non-Coding Genome—The ‘Dark Matter’ of Gene Regulation

Next-generation sequencing (NGS) has revealed that whilst 70% of the human genome is actively-transcribed, only 1–2% is protein-coding. However, almost all drug-discovery efforts to date are within the protein-coding space [10]. Thus, non-coding RNAs (ncRNAs), the major constituent of the human genome, and its capacity as drug targets to treat human pathologies, has largely been ignored. ncRNAs comprise microRNA (miRNA), long non-coding RNA (lncRNA), and other small RNA molecules. Since the discovery of the first miRNA (lin-4) in *Caenorhabditis elegans* in 1993 [11] and the first lncRNA (H19) in 1990 [12], the importance of the non-coding transcriptome in regulating gene expression, determining cell fate and driving pathogenic processes has become increasingly apparent. ncRNAs are also emerging as modulators of DNA damage response (DDR) [13,14,15,16]. Given the current focus on DDR pathway components as targets for PCa drug development, here we provide an up-to-date summary of the mechanisms and pathogenic consequences of lncRNA:miRNA crosstalk in PCa, with a specific focus on DDR. We also discuss the promises and challenges of using miRNA and lncRNA as PCa therapeutic targets and biomarkers.

## 2. MicroRNA Biogenesis and Function

The canonical miRNA biogenesis pathway starts with transcription of long mono- or polycistronic primary transcripts from their genes, named pri-miRNAs. Pri-miRNAs are further processed into precursor miRNAs (pre-miRNAs), 70-nucleotide-long hairpin structures, in the nucleus by the ribonuclease III enzyme, Drosha and its binding partner, RNA binding protein DiGeorge Syndrome Critical Region 8 (DGCR8) [17]. Pre-miRNAs are exported to the cytoplasm by an exportin (XPO5)/RanGTP complex and processed by the RNase III endonuclease Dicer to produce a ~22 nucleotide-long miRNA duplex. Duplex unwinding leads to loading of the mature miR (either 5p or 3p strand) onto the Argonaute (AGO)-containing RNA-induced silencing complex (RISC). AGO proteins coordinate downstream target gene silencing through interaction with other protein factors such as deadenylases, nucleases and translation factors [18]. The mechanism of miRNA regulation of gene expression at post-transcriptional level is through complementary base pairing of the miR “seed sequence” to the target RNA, most frequently in the 3′-untranslated region (3′-UTR) although 5′-UTR, coding sequence, and promoter binding can also occur [19]. This usually leads to translational suppression or transcript degradation mediated by RISC [20].

## 3. Long Non-Coding RNA Biogenesis and Function

LncRNAs are defined as RNA transcripts longer than 200 nucleotides and lacking coding potential, although some lncRNAs have been found to contain short open reading frames (sORF), which can encode micropeptides or small proteins [21]. Similar to mRNA, canonical biogenesis of lncRNAs involves transcription by RNA polymerase II [22]; some lncRNAs are then spliced, with the majority 5′ capped and 3′ polyadenylated [23]. Different classes of lncRNAs are transcribed from different DNA elements; based on their transcriptional origin, they can be sub-categorized into sense, antisense, intronic, intergenic, bidirectional, promoter-associated and enhancer-associated lncRNAs [24,25]. Most lncRNAs are poorly conserved between species and expressed at relatively low basal levels compared with protein-coding mRNAs. LncRNAs also show tissue- or cell type-specific expression and diverse subcellular distribution patterns, many being primarily nuclear, in contrast to exclusively cytosolic mRNAs [26,27,28]. Subcellular localization of lncRNAs is a key determinant of their biological functions and regulatory mechanisms. Nuclear, cytoplasmic and mixed distribution patterns have been observed. Importantly, understanding the localization of lncRNA is critical in the choice of method for manipulatinge lncRNA levels in vitro or in vivo, which is a critical step in the development of lncRNA-based therapeutics [29]. In general, nuclear lncRNAs function as modulators of gene expression at the epigenetic and transcriptional level in *cis* or *trans* through a number of mechanisms, including as signals, decoys, guides, scaffolds and enhancers [30].

**Figure 1:**
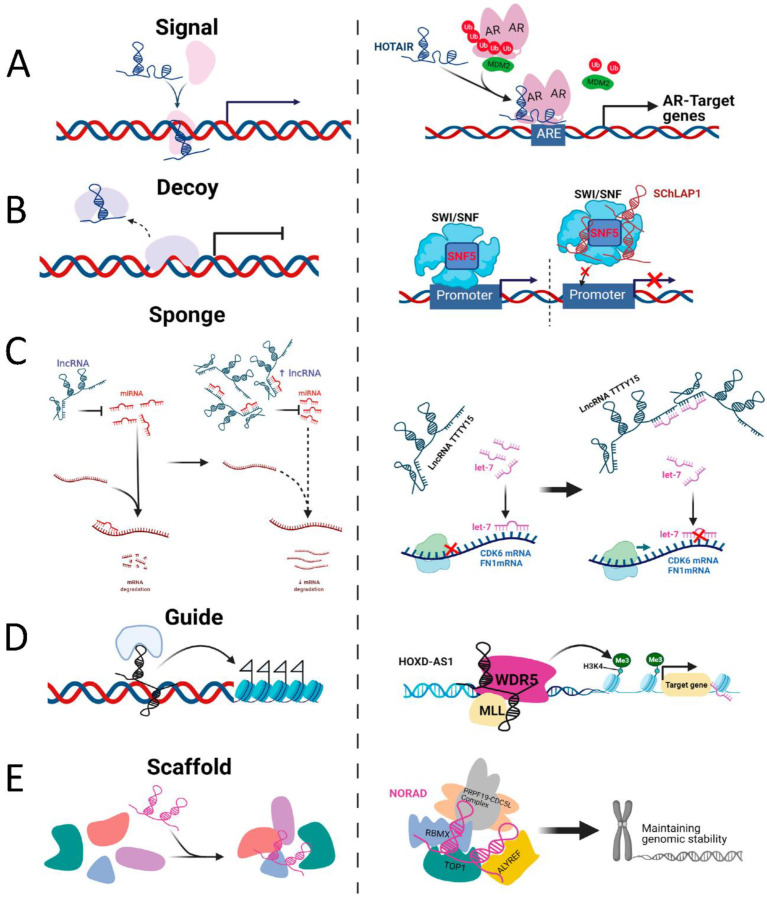
Schematic diagram of the molecular mechanisms of four lncRNA archetypes and their examples. (**A**) as signals, lncRNAs regulate transcriptional activity or gene expression (e.g., lncRNA HOTAIR). (**B**) as decoys, lncRNAs can bind transcription factors or regulatory proteins and displace them from DNA binding sites (e.g., lncRNA SChLAP1). (**C**) as sponges, lncRNAs can function as miRNA sponges and compete for miRNA binding to its target mRNA expression (e.g., lncRNA TTTY15). (**D**) as guides, lncRNA can recruit or relocalise regulation factors to activate or repress gene expression either in “*cis*” or “*trans*”. (e.g., lncRNA HOXD-AS1). (**E**) as scaffolds, lncRNAs can act as adaptors, bringing binding partner proteins within close proximity to aid the formation of Ribonucleoprotein complexes (e.g., lncRNA NORAD).

As signals, lncRNAs regulate transcriptional activity or gene expression. For example, the (androgen-downregulated) lncRNA HOTAIR can bind to the transcription factor androgen receptor (AR), to block its interaction with the E3 ubiquitin ligase MDM2 and prevent AR ubiquitination and protein degradation [31]. As decoys, lncRNAs can bind transcription factors or regulatory proteins and displace them from DNA binding sites. For instance, the lncRNA SChLAP1 (second chromosome locus associated with prostate-1) directly binds to SNF, preventing the SWI/SNF complex from binding to target promoters and leading to repression of target gene expression [32]. As guides, lncRNA can recruit or relocalise regulation factors to activate or repress gene expression either in “*cis*” or “*trans*”. An example is lncRNA HOXD-AS1, which recruits WDR5, a component of MLL1 complex, to directly interact with the promoter region of target genes and promote gene expression by mediating H3K4me3 [33]. As scaffolds, lncRNAs can act as adaptors, bringing binding partner proteins within close proximity to aid the formation of Ribonucleoprotein complexes. An example is the interaction of lncRNA NORAD with the DNA-damage response component, RBMX, to assemble topoisomerase complex NARC1, which contributes to the maintenance of genomic stability [34] (Figure 1).

Cytoplasmic lncRNAs function principally to modulate mRNA stability and translation. One of the important ways in which they achieve this is as competitive endogenous RNAs (ceRNAs) [35], which can impair miRNA activity through sequestration, thereby derepressing other miRNA targets [36,37] and regulating a wide range of biological processes [38].

## 4. LncRNA-miRNA Interactions

The regulatory mechanisms and functions of non-coding transcripts are increasingly revealing novel insight into the physiological and pathological processes of different diseases, including cancer [39,40,41,42]. RNA-RNA interactions exert regulatory functions within complex cellular networks, fine-tuning gene activity and permitting exquisitely-controlled environmental responses [43].

One well-characterised regulatory RNA-RNA interaction is miRNA targeting. Almost all RNA species, including small non-coding RNAs, pseudogenes, lncRNAs and circular RNA (circRNAs) contain miRNA recognition elements (MREs) that determine RISC-bound miRNA association and, in most cases, target inhibition. mRNAs and lncRNAs frequently contain multiple MREs, and each miR targets potentially hundreds of transcripts. An important corollary of these dynamic regulatory networks of interactions is the ability of lncRNA to act as molecular decoys or sponges to regulate miRNA activity, and by extension, the ability of different miRNA target transcript MREs to ‘compete’ for miRNA binding, resulting in de-repression of other targets [44,45,46,47]. This phenomenon, dubbed the ‘competitive endogenous RNA (ceRNA)’ hypothesis, was first described by Poliseno et al., who showed that certain pseudogene transcripts are biologically active units with miRNA-decoy function; retaining many miRNA binding sites, these can competitively bind with many miRNAs, acting as “perfect decoys” for their ancestral genes [48]. This decoy mechanism likely extends beyond pseudogenes to include other long noncoding and protein-coding transcripts. Indeed, Salmena et al. later demonstrated that lncRNAs, mRNAs and pseudogenes can act as ceRNAs within large-scale regulatory networks, using MREs as regulation ‘language’ [44]. The ceRNA phenomenon is exploited in the use of miR ‘sponges’ in functional investigations; artificial transcripts containing dozens of binding sites for the same miRNA in tandem under the control of a strong promoter are transfected into cells to effectively reduce the cellular pool of miRNAs [49].

**Figure 2:**
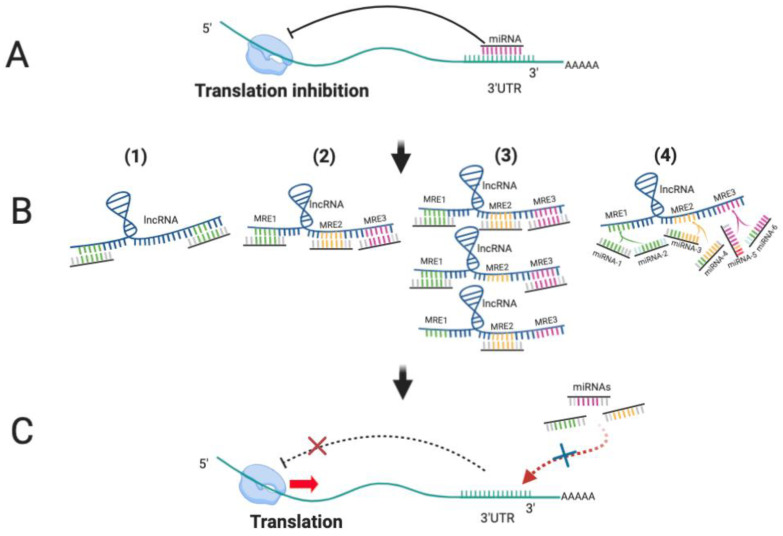
LncRNAs function as competing endogenous RNAs (ceRNAs) to sponge miRNAs. (**A**) miRNAs bind to the 3′UTR of their target mRNAs to block translation; (**B**) (1) LncRNAs display complete or partial complementary with miRNAs; (2) LncRNA containing multiple MREs can sequester multiple miRNAs; (3) The increased expression of lncRNAs leads to more binding to miRNAs, resulting in fewer miRNA molecules to bind to other target mRNAs; (4) Different miRNAs bind to lncRNA through same MREs, leading to competition for binding sites. (**C**) As lncRNAs function as ceRNA to sequester miRNAs away from other target RNA, translation of targets is derepressed.

The effectiveness of lncRNAs as ceRNA will be dependent on abundance of miRNA and its target transcripts, their subcellular localization, levels of specific RNA binding proteins (RBPs), as well as the relative binding affinities of the miRNA for different MREs. Since many lncRNAs are expressed at low levels, this may negatively impact their ceRNA capacity [39]. However, their length means that they could potentially ‘fine-tune’ activity of multiple miRNAs (Figure 2).

## 5. LncRNA as Oncogenic ceRNAs and MiR Sponges

The majority of described lncRNAs in PCa have been experimentally demonstrated to function as oncogenes, often growth-promoting and increased in prostate tumours compared to benign tissue, here the best characterised are summarised.

The well-known PCa tumour suppressor gene, *PTEN*, is regulated by ceRNA activity of its non-coding pseudogene, *PTENP1*, which competes for binding of regulatory miRNAs such as miR-19b, miR-21, miR-26a and miR-214 [48]. Another example is PlncRNA-1 (prostate cancer-up-regulated long noncoding RNA), which is increased in PCa and regulated by AR. Functionally, PlncRNA-1 upregulation induces PCa cell proliferation and epithelial-mesenchymal transition and represses apoptosis [50,51,52]. Mechanically, PlncRNA-1 functions as ceRNA to sponge AR-targeting miRNAs, miR-34c and miR-297, in both in vitro and LNCaP xenograft in vivo models [52]. Another proposed mechanism by which the above-mentioned SChLAP1 promotes aggressive PCa growth is by acting as ceRNA for miR-198, resulting in activation of the MAPK1 signalling pathway [53]. Further PCa-relevant interactions are shown in Table 1.

The lncRNA MALAT1, shows dysregulated expression across multiple cancers, including lung cancer [54] and breast cancer [55]. Similarly, in PCa, MALAT1 shows upregulation during cancer progression and is positively correlated with PSA, Gleason score and tumour stage [56]. Silencing of MALAT1 inhibited PCa cell proliferation, migration, invasion, epithelial-mesenchymal transition (EMT) and promoted cell apoptosis, even in xenografts models [57,58,59]. Of note, MALAT1 expression levels were increased in docetaxel (DTX)-resistant AR-negative PC3 and DU-145 cells, and DTX-resistant PCa patient tumours, and its overexpression enhanced DTX-chemoresistance in vivo. Mechanically, MALAT1 was shown to sponge miR-145-5p to derepress the miR-145-5p target, AKAP12. Both miR-145-5p overexpression and AKAP12 silencing rescued effects of MALAT1 on tumourigenic processes and DTX resistance [60]. MALAT1 has also been shown to act as ceRNA for miR-1, derepressing its oncogenic target KRAS in AR-negative PCa cells, and CORO1C in AR-positive PCa cells, respectively [57]. Importantly, there are links between oncogenic AR activity and MALAT1 in PCa: dihydrotestosterone (DHT) stimulation significantly induced MALAT1 in vitro, and MALAT1 was shown to act as a ceRNA for AR through competing for AR-targeting miR-320b. In vivo, MALAT1 knockdown suppressed tumorigenic and metastatic capacity of PCa xenografts [59]. MALAT1 also shows promise as a diagnostic urinary biomarker of PCa [56,59,61]. The relative importance of MALAT1’s diverse modes of action are difficult to dissect, but since it has been shown to be one of the most frequently miR-associated transcripts in PCa (AGO-PAR-CLIP-seq identifies interactions with 600 different miRNAs in PCa cell lines) [62], its potential for therapeutic targeting may be limited by its complex interactome and anticipated broad effects of inhibition.

Expression of another lncRNA with oncogenic properties, nuclear-enriched abundant transcript 1 (NEAT1), was significantly increased in PCa tumour versus benign tissues and elevated in DTX-resistant versus-responsive tumour samples [63,64]. Promotion of chemo-resistance is achieved, at least in-part, through NEAT1 function as a ceRNA to derepress ASCL4, RET and HMAG1 by binding miR-34a-5p, miR-204-5p and miR-98-5p, respectively [63,64].

LncRNA urothelial carcinoma associated 1 (UCA1) was first identified in bladder cancer [65]. However, UCA1 is also positively correlated with Gleason score, advanced TNM (tumour/node/metastasis) stage and shorter overall survival of PCa patients [66,67]. Similar in its mode of action to MALAT1 and NEAT1, LncRNA UCA1 upregulates cancer-promoting Sirt1, CXCR4, and activating transcription factor-2 (ATF-2) through sponging of miR-204. Silencing of UCA1 inhibited PCa cell proliferation, migration and invasion and promoted chemo-sensitivity in vitro and tumour growth in vivo [66,68,69].In addition, UCA1 sponges tumour-suppressive, anti-proliferative miR-143, leading to derepression of its oncogenic target, MYO6 in PCa [70]. It also regulates PCa cell apoptosis is through depression of apoptotic regulator, Bcl-2 by sponging Bcl-2-targeting miR-184 [71].

LncRNA Taurine-upregulated gene 1 (TUG1) was first discovered for its essentiality in the developing rodent retina [72]. Its dysregulation has been reported to have both oncogenic and tumour suppressive activity, depending on the context [73]. In PCa, TUG1 expression is increased in cancerous versus benign prostate tissue, and high TUG1 expression is correlated with reduced survival and poor PCa prognosis [74,75]. TUG1 was shown to act as ceRNA to sponge miR-26a and miR-496, promoting PCa cell proliferation, migration, invasion and EMT [76,77]. Its silencing repressed DU145 xenograft tumour growth and enhanced radiosensitivity in vivo by upregulating miR-496 and inactivating Wnt/b-catenin signalling through inhibiting expression of β-catenin, cyclin D1 and c-myc. In addition, miR-496 inhibition alleviated the inhibitory effects of TUG1 knockdown on repression of β-catenin, cyclin D1 and c-myc expression [76]. It has also been shown that TUG1 enhances SMC1A expression via sponging miR-139-5p [75].

PCA3 is highly-expressed, PCa-specific lncRNA, that can activate AR signalling to promote cell survival. It has also been approved by the Food and Drug Administration (FDA) in the USA as a diagnostic biomarker of PCa [78]. Microarray analysis identified PCA3 is increased in PCa patient tumours compared to adjacent benign tissues. In order to understand molecular mechanisms of PCA3 action, transcription factor (TF) promoter binding profiling arrays were carried out, identifying Snail as a direct promoter-binding activator of PCA3 expression. This is important as Snail is elevated in mCRPC and is required for hypoxia-induced PCa cell invasion and may be an informative biomarker of recurrence [79]. In a similar mechanism of action to the above lncRNAs, PCA3 act as ceRNA to sponge miR-1261 and derepress PRKD3 (protein kinase D3) to promote invasion and migration in PCa [79] shRNA-mediated PCA3 knockdown effectively repressed the cell proliferation, invasion, migration and induced autophagy in vitro, and inhibited the tumour growth of LNCaP xenografts in vivo [79]. Bioinformatic analysis and RNA immunoprecipitation identified miR-218-5p binding sites within PCA3 in PCa. miR-218-5p: PCA3 binding, resulted in loss of miR-218-5p tumour suppressor activity [80,81,82,83]. Indeed, silencing of PCA3 inhibited cell proliferation and migration, and induced apoptosis through increased miR-218-5p activity. It is suggested that miR-218-5p tumour suppressive effects are mediated via targeting of HMGB1. In vivo, shRNA mediated knockdown of PCA3 significantly inhibited tumour growth of PC3 xenografts and reversed the oncogenic effect of antagomir inhibition of miR-218-5p. Thus, PCA3 acts as a sponge of miR-218-5p and regulates HMGB1 to facilitate PCa progression [84].

Also functioning as a ceRNA through miR-218-5p binding is the lncRNA, lncAPP (lncRNA activated in PCa progression), identified from RNA-seq analysis of 65 prostate tumours and matched adjacent normal tissues. LncAPP is elevated in PCa tissues and urine samples from locally advanced/metastatic PCa patients compared with patients with localised disease. It is also correlated with PCa progression, suggesting that lncAPP could serve as a potential biomarker for the progression of PCa. LncAPP induced PCa cell proliferation, migration, invasion and EMT process in vitro. Overexpression and knockdown of lncAPP significantly promoted and inhibited tumour aggression, respectively. The underlying mechanism is that lncAPP competitively binding miR-218 to facilitate derepression of ZEB2/CDH2 [85], which suggest that lncAPP-miR-218-ZEB2/CDH2 axis plays a vital role in PCa progression and serve as a potential therapeutic target.

Further studies have found that lncRNA small nucleolar host gene 12 (SNHG12) expression levels were significantly increased in PCa tissue samples compared with adjacent normal tissues. The high expression of SNHG12 positively correlated with PSA, Gleason score, lymph node metastasis, and advanced residual tumour grade, as well as poor prognosis of PCa patients, suggesting potential utility as a PCa prognostic biomarker [86,87,88]. Inhibition of SNHG12 repressed PCa cell proliferation, invasion, migration and promoted apoptosis and autophagy in vitro, as well as suppressing tumour growth in vivo. In a similar mechanism of action to other PCa-implicated lncRNAs, SNHG12 acts as a sponge of miR-195, to enhance Wnt signalling by increasing levels of β-catenin, cyclin D1 and c-Myc [87]. It also derepresses CCNE1 expression to activate the PI3K/AKT/mTOR signalling pathway [86]. In addition, SNHG12 also act as ceRNA to target miR-133b to accelerate the tumorigenesis of PCa [88].

In a study aimed at identifying risk alleles amongst PC patients with aggressive disease, and in men with a strong family history of PC, PCAT-1 was shown to be associated with increased risk of PC [89]. This may be due to its interaction with the oncogene, c-MYC. C-MYC is highly expressed in PC. PCAT-1 has been shown to upregulate and stabilize c-MYC post-transcriptionally and abrogate its downregulation by miR-34, thereby increasing cell proliferation capacity of this oncogene. PCAT-1 was shown to stabilise the c-MYC transcript through association with c-MYC 3′UTR [90]. Enhanced oncogenic activity of both c-MYC and PCAT-1 in PC may be additionally attributed to the rs72725854-habouring enhancer present in a non-coding region of the 8q24 locus which gains enhancer activity in PC cell lines and tumours, but not in normal prostate tissues [91]. This androgen responsive enhancer is demonstrated to regulate PCAT-1 and MYC [91]. Guo and colleagues show that in PC, the MYC gene is regulated by a prostate-specific super enhancer overlapping the PCAT1 gene. Androgens can repress MYC expression by interfering with the interaction between MYC promoter and the super enhancer which may have an implication in the development of castrate resistant disease [92].

## 6. LncRNA as Tumour Suppressors

In contrast to the above-described oncogenic roles of lncRNA, several lncRNAs act as tumour suppressors to inhibit proliferation and migration, activate apoptosis, maintain genomic stability, and induce activity of well-established tumour suppressor signalling pathways [93].

One example is the lncRNA MEG3, which promotes growth inhibition, likely as a result of increasing levels of the tumour suppressor p53 protein. This is at least in part via its downregulation of mouse double minute 2 homolog (MDM2), which promotes p53 degradation [94,95]. MEG3 acts as ceRNA for a number of miRNAs [96]; its sponging of miR-9-5p derepresses QKI-5 expression to inhibit cell proliferation, migration, invasion and in vivo xenograft tumour growth in Pca [97]. Consistent with its tumour suppressor role, MEG3 expression levels were significantly decreased in Pca tumour tissues compared with adjacent tissues [97,98].

Growth arrest-specific 5 (GAS5) is another well-characterized tumour suppressive lncRNA. It is downregulated in cancers including Pca [99] and the gene for human GAS5 is within 1q25, a risk locus for sporadic and inherited forms of Pca [100]. GAS5 transcription is controlled by the mTOR (mammalian target of rapamycin) pathway [101,102] and the transcript accumulates in growth-arrested cells due to its role in coupling the nonsense-mediated decay (NMD) and mTOR pathways [101,103].In actively-proliferating and high mTOR activity cells, GAS5 transcription is increased [104] leading to NMD [105], which keeps GAS5 at low levels by degradation of GAS5 transcript [103]. On the contrary, suppression of mTOR activity, such as rapamycin treatment, results in cell growth inhibition and prevents the translation of GAS5 transcripts and degradation by NMD, which leads to increased expression of GAS5 [101]. In Pca, mTOR inhibitor-enhanced GAS5 expression in androgen-sensitive cell lines, and GAS5 silencing induced resistance to cytostatic effects of mTOR inhibitors in Pca cells [106]. A new mechanism has revealed that up-regulation of GAS5 can inhibit AKT/mTOR signaling through its direct target miR-103, to suppress cell proliferation, invasion, and migration. The tumour suppressive, AKT/mTOR-regulating role of GAS5 in Pca was further confirmed by in vivo xenograft model. In addition, expression of GAS5 RNA in Pca tissue was inversely correlated with clinical features including PSA level, Gleason grade, and pathological stage, suggesting that GAS5 loss may serve as a biomarker for Pca progression and GAS5-ATK/mTOR pathway is a potential therapeutic target for the treatment of Pca [107].

The X-inactive-specific transcript (XIST) is one of the first identified and well-characterized lncRNAs, functioning both as an oncogene and tumour suppressor in different cancers [108]. XIST expression was significantly down-regulated in Pca tissues, and further decreased in metastases; low XIST correlated with poor prognosis and increased clinical stage, presence of metastases, increased Gleason score, and PSA levels. Overexpression of XIST suppressed cell proliferation, metastasis and tumour growth both in vitro and in vivo. Mechanistic study revealed that XIST positively regulates Raf kinase inhibitor protein (RKIP) expression at the post-transcriptional level by sponging miR-23a [109].

A novel lncRNA LSAMP-AS1, which is an antisense to the mRNA encoding limbic system-associated membrane protein (LSAMP), was first identified for its association with senescence [110]. Recurrent deletion of chromosome 3q13.31, centering on the LSAMP locus, was prevalent in Pca tumours from African American men compared with Caucasian American men and associated with rapid disease progression, suggesting the involvement of LSAMP in the pathogenesis of Pca [111]. Antisense lncRNAs are transcribed from the opposite strand of a protein-coding genes and can act in *cis* to positively or negatively regulate expression of their overlapping protein-coding genes through diverse transcription-dependent mechanisms; they can also act in *trans* to regulate the expression of other genes [112]. This suggests that LSAMP-AS1 as well as LSAMP may play a vital role in Pca. A recent study addressed this hypothesis [113]: the authors found that LSAMP-AS1 expression levels were significantly decreased in Pca in two independent microarray datasets from benign and prostate cancer tissues (GSE55945 and GSE46602) and this was further validated in a different cohort [113]. In addition, low expression of LSAMP-AS1 correlated with poor overall and disease-free survival in Pca patients. Mechanistically, this study identified an important trans regulatory role of LSAMP-AS1 in Pca, namely upregulation of the tumour suppressor, Decorin (*DCN*), gene transcript by sponging miR-183-5p [113]. However, given the important roles of both LSAMP and LSAMP-AS1, it is important to delineate the precise regulatory relationship between the two transcripts, and how this is altered in Pca.

The lncRNA CASC2 (cancer susceptibility candidate 2) was found to act as a tumour suppressor in Pca through sponging of miR-183-5p, derepressing the miR-183-5p direct target SPRY2 (*Sprouty2*) [114]. Expression of SPRY2 has also been reported as downregulated in Pca and positively correlated with expression of lncRNA CASC2 [114,115]. In keeping with this, lncRNA CASC2 and SPRY2 were found to be down regulated while miR-183-5p was significantly upregulated in Pca tissues compared with adjacent benign tissues, and the down- and up-regulation respectively correlated with higher PSA levels, Gleason score, presence of metastases and shorter overall survival [114,116,117,118,119,120]. Of note, another study found that the miR-183-5p passenger strand, miR-183-3p, was down-regulated in Pca tissues and targets HMGN5 to repress cell proliferation, migration and apoptosis [121]. The opposing expression profiles and purported activities of the two miR duplex strands may reflect altered relative incorporation of the two strands into the AGO2-containing RISC during Pca progression. Further mechanistic studies are needed to investigate this and its consequences for lncRNA CASC2 modulation of SPRY2.

## 7. Non-Coding RNAs in DNA Damage Response

Our DNA is constantly exposed to various exogenous and endogenous insults that cause damage to the DNA either as single strand break (SSBs) or the more deleterious double strand breaks (DSBs) [122]. If unrepaired, such aberrations can result in gene mutations, chromosome rearrangement, genomic instability, chromothripsis and the onset and progression of cancer. In response to DNA damage, eukaryotes have evolved different mechanisms which requires diverse array of proteins to sense the damage, transduce damage signals and efficiently repair lesions [123]. These mechanisms are collectively termed the DNA damage response (DDR), which additionally stops cell cycle progression until the damaged DNA is repaired [124].

Base excision repair (BER), nucleotide excision repair (NER) and mismatch repair (MMR) are SSBs repair pathways. Each pathway has its unique damage recognition step, regulating proteins and varying fidelity. BER is initiated by DNA glycosylases and it corrects base lesions which do not significantly alter the structure of DNA. It is a rapid and efficient pathway as repair is limited to a damaged base [125]. Bulky adducts produced on DNA as a result of exposure to UV light or chemical agents cause distortion to the DNA helix. These lesions are removed and the DNA repaired via the NER pathway which is made up of the transcription-coupled NER and global genomic NER. The XPA-RPA and XPC-HR23B are two protein complexes that recognises damage and the efficiency of this pathway is dependent on the degree of distortion on the DNA helix [126,127]. Errors such as small insertions, deletions and mis-paired bases are corrected by the MMR pathway. The efficiency of this pathway varies depending on the location of the lesion in the genome (reviewed in [128]) however Edelbrock et al. showed that during DNA synthesis in normal physiology, MMR functions at increased efficiency with a high fidelity of repair during DNA synthesis [129]. Unrepaired SSBs lead to DSBs which are repaired via homologous recombination (HR) and non-homologous end joining (NHEJ). The repair pathway of choice is influenced by the phase of the cell cycle.

## 8. Role of DNA Damage Induced Non-Coding RNAs in DDR

The roles of the protein constituents of the DDR pathways are relatively well-characterised, however there are emerging evidence that support key roles for ncRNAs in these processes. There is evidence that ncRNA synthesized in the vicinity of DNA damage play a role in repair. They can also act epigenetically or post-transcriptionally to modify DDR protein activity directly and may also modulate DDR pathway activation through regulation of transcription and DNA replication.

Using deep sequencing analysis, Wei et al. demonstrated that a class of small RNAs—termed DSB-induced small RNAs (diRNAs)—are produced from sense and antisense strands of the sequence close to the damage site following DSB induction in human cells [130]. Following damage, it is proposed that the sensor MRN complex recognizes the lesion, recruits RNA Polymerase II and the pre-initiation complex to promote transcription from the damage site. This generates damage-induced lncRNAs which are processed by DICER and DROSHA to produce diRNAs [131,132]. Michelini et al. also demonstrated the requirement of RNA polymerase II-dependent transcription in the recruitment of diRNAs and in the activation and regulation of DDR foci. They further showed that 53BP1, an indispensable component and regulator of DDR, associates with diRNAs and damage-induced long non-coding RNAs [133]. DSBs that occur in the repetitive ribosomal DNA also induce the biogenesis of diRNAs [134]. Bonath and colleagues revealed that there are at least two sub-population of diRNA; one small RNA population (21-22nt) is dependent on DICER and has a 5′ uracil bias, whereas the second group is heterogenous in length with a characteristic guanine bias at the 3′ end. In contrast to the above studies, these data showed that DROSHA is not necessary for diRNA generation, and only one of two classes of diRNA require DICER processing [134].

Whilst there are conflicting reports on the role of DROSHA and DICER in the processing and maturation of diRNAs that maybe context dependent, additional roles in recruitment of DDR factors have been described. DNA damage leads to the phosphorylation of DICER resulting in its nuclear accumulation and recruitment to DSB sites, where it processes nuclear dsRNA to promote repair [135]. Knockdown of DICER impaired recruitment of 53BP1 and MDC1 to damage foci, corroborating the requirement of DICER in DDR [135]. On the other hand, there is evidence to support DROSHA’s recruitment to DSBs by the MRN complex. It is also purported to associate with DSBs in a transcription-independent manner to preferentially promote NHEJ repair as against HR [136].

It has also been demonstrated that diRNAs can promote recruitment of DSB repair complexes to damage sites through AGO2 [130]. It was revealed that AGO2 interacts with RAD51—a highly conserved protein which is indispensable in HR. RAD51 accumulation at DSBs was shown to be dependent on small-RNA binding and catalytic activity of AGO2 was required for RAD51 HR repair [133]. The authors proposed that the recruitment of RAD51 to DSB sites is guided by diRNAs through its interaction with AGO2 [133].

Chromatin remodelling, a feature of DSB repair, promotes recruitment of repair factors to damage site [137]. Through AGO2, diRNAs interact with the chromatin modifying enzymes acetyltransferase, Tip60 and methyltransferase, MMSET [138]. This interaction guides the recruitment of both enzymes to DSBs, where the chromatin assumes an open and flexible configuration which facilitates access of BRCA1 and RAD51 to damage sites promoting HR [138].

Non-Coding RNA Activated by DNA Damage, NORAD, a highly-expressed, evolutionarily-conserved lncRNA was discovered in the colon cancer cell line, HCT116, following its p53-dependent induction upon DNA damage [139]. NORAD was shown to sequester PUM1 and PUM2 proteins, which are responsible for turnover of DNA repair transcripts. Indeed, genes regulated by PUM1/2 are sensitive to NORAD manipulation [139]. SAM68, a RNA binding protein is an interaction partner for NORAD and PUM2 and it plays a role in the regulation of PUM proteins by NORAD, chromosome segregation and progression through mitosis by buffering the sequestering activity of PUM proteins [140]. Overexpression of NORAD derepresses PUM1/2 target genes with roles in chromosomal integrity, DNA replication and DDR, and thus it is thought to be required for maintenance of genome stability [141,142]. In a bid to elucidate the molecular mechanism of NORAD, Munschauer et al. demonstrated that NORAD is also crucial for the assembly for a ribonucleoprotein complex which physically connects proteins with prominent roles in DNA replication and repair [34]. This complex, referred to as NORAD activated ribonucleoprotein complex (NARC1), is made up of RBMX, PRPF19, CDC5L, TOP1 and ALYREF [34], which are prominent DDR proteins. This predominantly cytoplasmic lncRNA is reported to be overexpressed and correlated with poor prognosis in colorectal, lung, gastric, bladder, thyroid, ovarian and oesophageal squamous cell carcinoma [143,144,145,146,147,148,149]. Zhang & Guo report lower cell proliferation, migration and higher apoptosis following silencing of NORAD in PC cell lines, although only AR-negative advanced metastatic models were used, and in vivo effects were not assessed [150]. As a ceRNA, NORAD enhances activity of E2F1, a transcription factor in HR repair, by acting as a decoy for its targeting miR, miR-136-5p in lung cancer [148,151]. It was also shown to sponge miR-608 to derepress FOXO6 and promote gastric cancer cell proliferation [143].

## 9. Role of lncRNA and miRNA in the Regulation of DNA Double Strand Breaks (DSB)

HR is the error-free repair mechanism of DSB repair which uses a sister chromatid as template for repair. It takes place in the S and G2 phase of the cell cycle. It delivers a high-fidelity repair of DSBs and one of its principal components is BRCA2 which mediates the recruitment of RAD51 to damage sites and protects the replication fork [152]. RAD51, a recombinase, is essential for homologous pairing and strand exchange in the repair of DSB. Overexpression of the lncRNA, PCAT-1, which is predominantly cytoplasmic, significantly reduced the stability of BRCA2 mRNA in the Pca cell line, DU145 [153]. This further decreased RAD51 foci formation, impaired HR and imparted high sensitivity to Olaparib, a PARP inhibitor. PCAT-1 was shown to directly repress the 3′UTR of BRCA2 post-transcriptionally and the 5′ terminus of PCAT-1 was required for the repression [153] highlighting the role of lncRNA in the integrity of repair pathway.

The long non-coding radiation induced, lnc-RI has also been reported to influence the HR process by regulating the stability of RAD51 [154]. Knockdown of lnc-RI resulted in an accumulation of DSBs characterized by an increase in gamma-H2AX foci as well as decreased RAD51 at both mRNA and protein levels. Lnc-RI had no effect on protein degradation but was shown to be necessary for RAD51 mRNA stabilization. Furthermore, it was demonstrated that miR-193a-3p interacted directly with RAD51 mRNA via its 3′UTR as well as lnc-RI and overexpression of this miRNA reduced the expression of both RAD51 and lnc-RI with a concomitant increase in gamma-H2AX foci. The authors concluded that lnc-RI plays a role in HR by regulating the stability of RAD51 mRNA by competitively binding with miR-193-3p thereby reducing its inhibition of RAD51 [154]. MiR-193a-3p overexpression inhibits cell proliferation and induces G1-S phase cell cycle arrest [155]. This may be mediated in part through its 3′UTR-directed repression of cyclin D1, an essential regulator of the G1-S transition [155]. It has been shown that cyclin D1 directly binds RAD51 and is recruited to DNA damage sites in a BRCA2-dependent manner, and that downregulation of cyclin D1 impairs recruitment of RAD51 thereby impeding HR [156]. Thus, it is possible that lnc-RI and miR-193a-3p converge upon RAD51 and cyclin D1 to modulate DDR-directed cell cycle progression via two independent connected pathways.

To maintain the integrity of the genome, DDR processes are tightly controlled by the cell cycle which is regulated by the activities of cyclin-dependent kinases (CDK). DNA damage that occurs during S-phase (repaired largely through the HR pathway) causes p53-dependent accumulation of p21 during G2 and G1 phases which inhibits the activities of CDK, thereby promoting cell cycle arrest, inhibition of cell proliferation, senescence and apoptosis [157].

The transcription factor cell division cycle 5 like, CDC5L, is part of the pre-mRNA processing complex and is a regulator of G2/M phase of the cell cycle. Its interaction with ATR is required for the activation of the S-phase checkpoint in response to stalling of the replication fork as well as activation of the downstream DDR effectors CHK1, Rad17 and FANCD2 [158,159]. CDC5L is regulated by the lncRNA, Nuclear Enriched Abundant Transcript, NEAT1, in PCa [160]. Li et al. show that NEAT1 and CDC5L colocalize partially in the nucleus and directly interact. Using a dual-luciferase reporter system, silencing of NEAT1 in the AR-null PCa cell lines, PC3 and DU145, suppressed CDC5L-mediated transcriptional activation, indicating that the activity of this transcription factor is dependent on the expression of NEAT1. NEAT1 loss-of-function led to DNA damage in PC3 and DU145 cell lines, characterized by γH2AX phosphorylation, and cell cycle arrest in the G1, G2 and M phases [160]. NEAT1 is reported to be overexpressed in PCa tissues and cell lines and it positively correlates with Gleason scores and metastatic staging [64]. In addition to its ability to promote DNA repair and cell cycle progression via CDC5L, NEAT1 promotes ATR signalling in response to DNA damage or replication stress and is involved in a negative feedback mechanism that decreases activation of p53 [161].

Following DSBs, ATM is auto-phosphorylated, leading to downstream phosphorylation of intermediates such as p53 and H2AX, which then activate cell cycle checkpoints and DNA repair, respectively. Wan and colleagues have shown that lncRNA, ANRIL, is transcriptionally upregulated via E2F1 following DNA damage in an ATM-dependant manner in the colorectal carcinoma cell line HCT116 [162]. In complex with CBX7, elevated ANRIL was shown to repress transcription from the p14, p15 and p16 cyclin-dependent kinase inhibitor-containing INK4B-ARF-INK4A locus (from which it is also transcribed in the anti-sense direction from an independent promoter) through recruitment of PRC-1 and PRC-2 during the late stage of DDR. The authors postulate that this functions to promote cell cycle progression following completion of break repair, indicating that ANRIL can inhibit cell cycle checkpoints to promote cell cycle progression [162]. Notably, ANRIL has also been shown to repress senescence in ovarian cancer [163]. Although there are no reports directly linking ANRIL, PCa and DNA damage, ANRIL is overexpressed in PCa tissues where it enhances cell proliferation and migration by regulating the let-7a/TGF-β/Smad1 pathway [163,164]. Downregulation of ANRIL inhibits tumorigenicity and enhances the cytotoxicity of the DNA damaging drug cisplatin by upregulating the expression of let-7a in ovarian [165] and nasopharyngeal cancers [166]. Thus, ANRIL may promote cell cycle progression and cell survival in response to DNA damage via both repression of CDKs and upregulation of let-7a, representing a promising potential therapeutic target.

The repair efficiency of HR is also influenced by the lncRNA, DNA damage sensitive RNA1 (DDSR1). Following DNA damage in U2OS, HCT116 and PC3 (bone, colon and PC cell lines respectively), DDSR1 was induced in an ATM-NFκB dependent manner [167]. Silencing of DDSR1 resulted in significant reduction in the expression of critical DDR proteins, gamma-H2AX, pRPA, pCHK1, p53 following DNA damage with camptothecin, indicating that it acts downstream of ATM. Mechanistically, DDRS1 was shown to interact with BRCA1 and the RNA-binding repair protein, hnRNPUL1. DDSR1:hnRNPUL1 interaction prevents the promiscuous association of BRCA1 with DNA breaks that is inhibitory to HR. Indeed, DDSR1 loss-of-function led to aberrant BRCA1 recruitment [167]. Taken together, this study highlights the role of the lncRNA, DDSR1 as a regulator of HR.

NHEJ does not require a homologous template and occurs throughout the cell cycle. It involves error-prone ligation of broken DNA-ends. DNA-PK, Ku70/80 are key players in the NHEJ DDR machinery. In addition to NHEJ, DNA-PK plays important roles in HR and in the immune system via the V(D)J and class switch recombination [168,169,170,171]. This serine/threonine protein kinase, which phosphorylates ATM and H2AX leading to the detection of DSBs, is made of a catalytic subunit and a Ku heterodimer which consists of the Ku70 and Ku80 subunits that also bind DSBs [168,169]. Liquid-chromatography tandem mass spectrometry identified ceRNA-functioning lncRNA, SNHG12, as an important DNA-PK binding partner via its domain 4 sequence [172]. Importantly, this interaction facilitates the ability of DNA-PKs to bind ku70 and Ku80, and to mediate DDR. As the knockdown of SNHG12 led to an increase in DNA damage, the authors concluded that this lncRNA plays a role in DNA-PK dependent DDR [173]. Although SNHG12-modulation of DDR was not assessed in PCa, its expression is increased in PCa tissues compared with matched normal tissues [87]. High expression of SNHG12 was correlated with an aggressive phenotype in patients evidenced by higher Gleason score and lymph node metastasis. SNHG12 was also reported to promote proliferation and invasion, suggestive of an important role in prostate tumourigenesis [87]. Further bioinformatic analysis and molecular assays indicated that SNHG12 may have oncogenic activity in PC through sponging of miR-195, which is purported to act in a tumour-suppressive manner [87,174].

## 10. Targeting DNA Damage Response Pathways for Prostate Cancer Therapy

The heterogeneity of mCRPC, whilst contributing to drug-resistance, also provides opportunities for PCa personalized medicine. Approximately one-third of mCRPC patients have coding mutations in established DNA damage repair (DDR) genes, providing a rationale for their therapeutic exploitation. For example, PARP inhibitors (PARPi) work by blocking PARP catalytic action in the repair of single-strand DNA breaks, and by trapping of PARP proteins on DNA. These demonstrate efficacy in patients with defects in HR such as BRCA1/2 deletion/inactivating mutations by functioning through synthetic lethality and complete loss of DNA break repair capacity. Clinical trials (TOPARP-B, PROFOUND, TRITON2, GALAHAD) are underway to define the HR aberrations that render tumours susceptible to PARPi, and to assess the therapeutic potential of targeting other DNA repair proteins, for example, ATR inhibitors in ATM-deficient PCa [175].

It is increasingly apparent that factors beyond HR coding gene aberrations, such as epigenomic alterations and non-coding factors can modulate response to DNA damage-targeting drugs, particularly since (i) PARPi BRCA1/2-mutant response rate is only approximately 50%, (ii) efficacy has been observed in patients lacking mutations in key HR genes, and iii) variability in response is seen in patients harboring ATM, CDK12, CHEK2, PALB2 mutations, amongst others [176,177]. It is hoped that ongoing studies and clinical trials in carefully stratified populations will reveal genomic/non-genomic biomarker signatures of PARPi response to improve patient survival and negate morbidities of ineffective treatment (so-called ‘BRCAness’ transcriptomic panels have been proposed), and that alternative DDR-targeting drugs will prove efficacious in the context of non-BRCA aberrations. Since these treatments also apply strong selection pressure, and BRCA2 reversion mutations have been observed [178] rendering the tumour resistant to PARPi, combinatorial approaches may also be warranted.

## 11. Exploiting Non-Coding RNAs Therapeutically in PCa

Given that ncRNAs are recognised to be versatile and important molecules in the regulation of genes, they potentially represent efficacious drug targets or therapeutics. A caveat to their use in therapeutics is instability due to the many ribonucleases that can initiate their degradation in vivo. However, chemical modification to their structure has been shown to improve stability, specificity and immunogenicity, as well as pharmaco-kinetic and -dynamic properties. Strategies for targeting of ncRNAs include antisense oligonucleotides (ASOs) which target complementary RNA by Watson-Crick base pairing rules with high affinity as well as specificity; their mechanisms of action include steric hinderance, RNA interference, splice modulation and ribonuclease H1-dependent degradation [179]. Therapeutic ncRNA molecules include Gapmers, which are chimeric single-stranded oligonucleotides containing a central stretch of deoxynucleotide monomers between modified RNAs (2′-*O*-methyl RNA or 2′-*O*-methoxyethyl RNA, locked nucleic acids or constrained ethyl nucleosides) that are capable of activating RNA degradation by RNASEH1 [180].

The expression and function of lncRNAs can be inhibited by antisense-based strategies, such as RNA interference (RNAi) by siRNAs, short hairpin RNA (shRNA) and GapmeRs. Of these different lncRNA inhibition techniques, siRNAs preferentially show effective targeting of cytoplasmic lncRNAs, whilst GapmeRs can enter the nucleus to target nuclear-enriched lncRNAs by introducing ribonuclease H-dependent cleavage. Combined use of GapmeRs and siRNAs can improve knockdown efficacy, especially for lncRNAs that localize to both cytoplasmic and nuclear compartments [181,182,183]. However, presence of cellular nucleases and foreign RNA-activated innate immune pathways, for example, Toll-like receptor (TLR) and RIG-1, may limit effective cellular uptake of such molecules [184].

In 2018, the FDA approved the first RNA therapeutic Patisiran (brand name Onpattro) which is a siRNA for the treatment of familial transthyretin-mediated amyloidosis [185].The antisense oligonucleotide Nusinersen, which acts by splice modulation, has also been approved by the European Medicines Agency for treatment of spinal muscular atrophy [186]. These advances expand the possibilities for routine clinical use of RNA-based drugs across a diversity of human diseases. Following several decades of extensive siRNA and miRNA-based research and discovery, there are a number of RNAi and oligonucleotide-based drugs targeting protein-coding mRNAs in clinical trials [187]. Further, clinical trials (Phase I/II) of miRNA-based drugs (either miRNA mimics for gain-of-function or miRNA inhibitors for loss-of-function) have been undertaken [188] and approximately 55 lncRNA-based clinical trials are underway or have completed [189].

## 12. Exploiting miRNA-Based Agents in PCa Therapy

Therapeutically, miRNAs can be exploited using either mimics, to ectopically increase expression of a specific miRNA, or antagomiRs which bind to endogenous miRNAs, sequestering and preventing them from interacting with target transcripts. In both cases chemically-modified forms such as phosphorothioate backbone, 2-methoxyethyl nucleotides and locked nucleic acids have improved their stability and specificity.

The majority of anti-androgens in clinical use for PC target the ligand binding domain (LBD) or the AR. Unfortunately, a major mechanism of resistance to such drugs is the emergence of constitutively-active AR-variants that lack the LBD. Hence major research efforts are centred on development of therapeutics targeting non-LBD regions of AR. Targeting the 3′-UTR of the AR using miRNAs may represent a promising strategy in this regard. Work from our laboratory demonstrates that miR-346, -361-3p, -197 modulate AR signalling through association with the 3′ end of the 6.9 kb AR 3′UTR to enhances transcript stability [190]. Inhibition of these miRNAs markedly reduced transcript and protein levels of both wild-type and variant AR, with concomitant decreases in target gene expression [190]. In addition, inhibition of these miRNAs reduced proliferation, increased apoptosis and sensitised cells to anti-androgen treatment. Further, miR-361-3p and -197-3p levels were enhanced by anti-androgen treatment of patient-derived xenografts, nominating them as potentially relevant drug targets in CRPC. The long length of the AR 3′UTR as compared to its coding region indicate an important contribution of miRNAs and RNA-binding proteins to its regulation, suggesting that therapeutics based on these may be particularly effective in controlling AR activity.

As the androgen signalling pathway remains functional in the progression of PCa, targeting the AR is a viable treatment option at all disease stages. One RNA therapeutic in development is the antisense oligonucleotide AZD5312, which is designed to target full length, mutated forms and splice-variant AR, preventing the translation of the AR protein by hybridizing with its mRNA. Administration of this ASO in mCRPC patients who have previously failed standard of care treatments demonstrated that it is well-tolerated with evidence of prostate specific antigen and circulating tumour cell decline in some patients [191].

Irradiation is one of the main interventions used in the treatment of PCa hence radiation-sensitizing strategies may improve patient outcomes. PCa cells treated with miR-744-3p and miR-890 mimetics prior to radiotherapy showed significant delay in the resolution of the DNA damage maker gamma-H2AX over a 24-h period compared to radiation-only cells [192]. MiR-890 pre-treatment also enhanced effects of radiotherapy in reducing in vivo tumour volume, as compared to negative control. The mechanism of action of miR-890 was pinned on its ability to reduce expression of DDR-implicated genes WEE1, KU80, XPC and MAD2L2 [192]. MiR-449a has also been shown to increase radiotherapy sensitivity in LNCaP cells and xenograft models, evidenced by G2/M cell cycle arrest, decreased cell viability and suppressed tumour growth. Its potential tumour suppressive activity was attributed to targeting of the 3′-UTR of the c-MYC oncogene [193]. Thus miR-890 and -499a mimetics may represent efficacious radiotherapy sensitisers in PCa.

Aside from principally inducing DNA damage, ionizing radiation is immunogenic. Tao et al. demonstrated that overexpression of miR-16 and -195 may increase radio-sensitivity in PCa cells by blocking the expression of the immune checkpoint, PDL1 and enhancing the proliferation of functional cytotoxic CD8+ T-cells. They further show that high levels of these microRNAs were positively correlated with biochemical recurrence-free survival [194].

Cells bearing mutations in BRCA1/2 are deficient in HR and thus confer sensitivity to PARP inhibitors, which have been approved for the treatment of CRPC. Much remains to be learnt about the subset of patients who will optimally respond to such treatment [177]. Mimics of miR-107 and -222 were shown to sensitise tumour cells to Olaparib, a PARP inhibitor, in ovarian cancer by repressing the expression of RAD51 [195]. MiR-107 is downregulated in PCa cells and tissues and its overexpression is demonstrated to inhibit proliferation and induce cell cycle arrest [196]. It is plausible that miR-107 mimetics administered to PCa patients can also increase sensitivity to PARP inhibitors and promote its tumour suppressor effects. This remains to be investigated.

In order to delineate the therapeutic potential of the cholesterol-conjugated antagomiRs, anti-miR-221 and -222 were injected intratumorally at day 0, 5, 9, a total of three injection per tumour in SCID mice [197]. The authors showed that the average fold volume increase of treated tumours was significantly reduced compared to control groups. Total RNA from treated excised tumours showed a persistent reduction in the expression of both miR-221 and -222 compared to control tumours. They further revealed that these antagomiRs can lower the expression of the target miRNAs for as long as 24 days whilst increasing the levels of p27, a tumour suppressor gene that also plays a role in DDR DDR [197,198].

## 13. Exploiting LncRNAs in PCa Therapy

After two-decades of discovery and extensive research of miRNAs in human disease, there are several miRNAs already under phase 2 clinical trials [188]. Clinical trials of drugs targeting lncRNAs have been initiated [93]. The lncRNA related plasmid therapy BC-819, which is a DNA plasmid carrying the gene for diphtheria toxin-A (DTA) under regulation of the promoter of lncRNA H19 is currently in Phase III clinical trials in patients with bladder cancer [199,200]. However, several strategies have been employed for lncRNA manipulation of lncRNA that show pre-clinical promise.

Having observed that the lncRNA, ARLNC1, is a vital survival factor in AR-dependent PCa, an ASO was developed to target the transcript in cells lines and mouse xenograft models. This reduced in vivo tumour growth [201] indicating that ARLNC1 may be a viable therapeutic target in AR-dependent PCa. ARLNC1 may also serve as a potential biomarker as it is significantly overexpressed in localized and metastatic PCa compared to benign tissues [201].

The inhibition of novel PCa lncRNA activated in metastatic PCa (lncAMPC) by siRNAs was successfully achieved in vitro and in vivo. lncAMPC, expressed both in the nucleus and cytoplasm, is significantly increased in PCa tumour tissues and preferentially upregulated in metastatic compared to localized disease. The silencing of lncAMPC results in reduced cell proliferation, migration and invasion and significantly suppressed xenograft tumour growth in immunodeficient mice. Mechanistically, lncAMPC regulates LIF expression by sponging miR-637 in cytoplasm and enhances LIFR transcription by decoying histone H1.2 away from the upstream sequence of LIFR gene in the nucleus. The upregulation of LIF/LIFR activated downstream signaling through the Jak/STAT, MAPK and PI3K pathways, which were repressed by siRNA-mediated lncAMPC knockdown in vitro and in vivo. The inhibition of lncAMPC decreased, whilst overexpression increased, expression of PD-L1 in the xenograft tumour tissues. PD-L1 expression also positively correlated with lncAMPC-activated LIF level [202].The authors suggest that the combination of targeted lncRNA therapy and immune checkpoint inhibitors may be an effective novel strategy for PCa treatment, although relevance of this in the context of the immunodeficient host used in their experiments is unclear. The above example notwithstanding, the numbers of pre-clinical studies using siRNAs/shRNAs to target lncRNAs are very limited, in part due to lack of efficient delivery methods and limited bioavailability of siRNAs in mammals [203].

The expression of the lncRNA, Testis-Specific Transcript Y-Linked 15, TTTY15, is increased in PCa tissues compared with paired control tissues. It has been shown to promote PCa progression by acting as ceRNA for let-7, leading to derepression of the let-7 target oncogenic genes, CDK6 and FN1. The transcription factor FOXA1 is an upstream positive regulator of TTTY15. Thus, FOXA1-TTTY15-let-7-CDK6/FN1 axis is reportedly involved in the disease progression of PCa. Using several anti-sense oligo- and CRISPR-based strategies for TTTY15 loss-of-function, it was shown that TTTY15 silencing inhibits cell proliferation, migration and invasion, warranting its further pre-clinical therapeutic investigation [204].

## 14. Limitations and Challenges of lncRNA Therapeutics

LncRNA targeting represents a powerful therapeutic strategy for personalised medicine, due in part to their cell/tissue specific expression patterns, diverse tools for manipulation and increasing evidence for disease-relevant functionality. However, several limitations and challenges remain. Firstly, low conservation of lncRNAs between human and rodents poses considerable challenges to mechanistic studies and pre-clinical therapeutic assessment. Secondly, the relatively low abundance of lncRNAs compared with protein-coding genes and predominant nuclear localisation of many lncRNAs presents difficulties in terms of delivery of targeting agents across the nuclear membrane. ASOs, which function effectively in the cell nucleus due to the nuclear enrichment of effector RNase H, may present a viable approach here. Thirdly, the diverse functions attributable to a single lncRNA across different pathologies, coupled with context-specific dysregulation, may necessitate tissue-specific delivery modes and may affect the target specificity of lncRNA therapeutics. Fourthly, detailed characterization of lncRNA structure, functional motifs, and interplay with protein/RNA interactors is required before targeting can be considered, in order to mitigate risks of side-effects and toxicity in non-target tissues. Indeed, many published studies are limited to in vitro investigations: development of genetically-engineered mouse models with tissue specific deletion of candidate lncRNAs will give powerful insight into tissue-specific lncRNA activity, and for lncRNAs that are restricted to humans, systemic or injected delivery of ASOs to patient-derived xenografts (PDXs) represent a valuable approach to pre-clinical evaluation. Further, since ribosome profiling has shown that some short open reading frames (ORFs) in lncRNAs encode micropeptides with pathological activity [205], it is important to robustly confirm the non-coding nature of target lncRNAs. Finally, as for many putative therapeutics, delivery to the centre of solid tumours such as those of the prostate, often with hostile hypoxic environments, is a major hurdle to overcome. Systemic delivery will exhibit rapid clearance from the blood and accumulation in the liver and kidney, significantly decreasing delivery efficiency.

For tumour suppressive lncRNAs, it is desirable to restore their in vitro and in vivo function through overexpression. This is often achieved in cell lines by using recombinant viral system including adenoviruses, lentiviruses and adeno-associated viruses (AAVs). RNA-guided endogenous CRISPR activation (CRISPRa) is another useful tool to overexpress the lncRNA from endogenous loci or promoter, which is especially important for cis-acting lncRNAs [206]. Adenovirus-mediated overexpression of tumour suppressor lncRNA XIST suppressed cellular proliferation and metastasis in PCa both in vitro and in vivo through sponging miR-23a to regulate RKIP expression [109]. Due to the high transduction rates and robust transgene expression of adenovirus, such delivery systems show therapeutic promise and are the subject of ongoing clinical trials [207]. Compared with the transient transduction of adenovirus, lentivirus-mediated delivery systems can integrate DNA into the host genome to achieve long-term expression of their therapeutic transgene. LncRNA FENDRR expression levels are significantly decreased in PCa tumours. The upregulation of FENDRR expression levels in PCa cells were induced by lentivirus transduction, which acts as a ceRNA sponging miR-18a-5p, leading to upregulation of miR-18a-5p target, RUNX1, to inhibit cell proliferation and induce apoptosis [208]. However, to date, all such studies have been performed in cell lines and the clinical utility of such approaches remains to be determined.

## 15. Conclusions

The first small-interfering RNA drug Patisiran to treat polyneuropathy was approved by the FDA in 2018 [209]. Whilst the approval of the Pfizer-BioNTech and Moderna mRNA-based COVID-19 vaccines in 2020 in response to the COVID-19 pandemic [210] has renewed interest in RNA-based therapeutics, unfortunately, miRNA-based drugs are yet to deliver on their therapeutic promise, with only a handful progressing to Phase I or II clinical trials. Their exploitation in this regard requires a complete understanding of their impacts on cancer-associated processes. It is increasingly well-appreciated that miRNA activity is regulated by interactions with lncRNAs within complex regulatory networks to exert exquisite control of gene expression. A major recent focus in the ncRNA field has been on DNA damage processes, driven at least in part by the observation that lncRNAs (and some miRNAs) can associate with DNA, and the recent clinical success of DDR-targeting synthetic lethality approaches for personalised medicine in PCa.

It is now clear that both miRNAs and lncRNAs can impact DDR processes directly and indirectly, and at multiple levels through such phenomena as miRNA targeting of DDR proteins or their regulators, lncRNA sequestration of DDR-impacting miRNAs and regulation of protein:chromatin interactions. Intriguing data from multiple groups have now clearly shown the generation of long and small ncRNAs from sites of DNA damage, whose DDR-promoting functions remain to be fully characterised.

In contrast to miRNAs, approaches for drug-targeting of lncRNAs are still in their infancy but show considerable promise due to the high tissue-specificity of these molecules, if technical hurdles can be overcome. Further, tissue-specificity makes them particularly attractive as candidate biomarkers. Indeed, lncRNA PCA3 has been approved by the FDA as a urine-based molecular diagnostic biomarker for PCa.

In summary, the successful application of ncRNA-based therapeutics requires further molecular, mechanistic and preclinical studies, as well as development of novel delivery modalities. Recent promising advancements in these areas suggest that the future is bright for ncRNA therapeutics in PCa.

**Table 1:** LncRNA: miRNA interactions in prostate cancer and their phenotypic implications.

LncRNA	Interacting miRNA	Target mRNA	Expression in PCa	Functions in PCa
SNHG14	miR-5590-3p [211]	YY1	Increase	Promote cell proliferation, invasion, and repress apoptosis
TUG1	miR-496 [76]	Wnt	Increase	Promote cell proliferation, migration, invasion, colony survival fraction and repress apoptosis
	miR-139-5p/miR-26a [75]	SMC1A		
SOX2-OT	miR-425-5p [212]	HMGB3	Increase	Promote cell proliferation, migration, cancer metastasis, and active the Wnt/b-catenin signaling pathway
	miR-369-3p [213]	CFL2		
SNHG1	miR-377-3p [214]	AKT2	Increase	Promote cell viability, growth, cell cycle progression and
	miR-199a-3p [215]	CDK7		suppress cell apoptosis
UCA1	miR-143/miR-204	MYO6/Bcl2	Increase	Promote cell growth, invasion, and suppress apoptosis
	miR-184 [66,68,69,70,71]	Sirt1/CXRC4		
		ATF2		
SNHG12	miR-195 [86,87]	CCNE1	Increase	Promote cell proliferation, invasion, migration, viability; Suppress apoptosis and autophagy; activate PI3K/AKT/mTOR pathway and Wnt/b signaling pathway
	miR-133b [88]			
NEAT1	miR-34a/miR-204 [63]	RET/ACSL4	Increase	Promote cell growth and invasion; potential prognostic biomarker
	miR-98 [64]	HMGA2		
DANCR	miR-34a [216]	JAG1	Increase	Promote cell proliferation, resistant to apoptosis; Silence of DANCR improved docetaxel and paclitaxel efficacy
	miR-135a [217]			
MALAT1	miR-1 [57,58]	KRAS/CORO1C	Increase	Promote cell proliferation, migration, invasion, EMT and resistant to apoptosis; promote tumor growth in vivo
	miR-145/miR-320B [59,60]	AKAP12/AR		
SNHG7	miR-324-3p [218]	WNT2B	Increase	Promote proliferation, migration, invasion, and EMT; induce cell cycle arrest; silence SNHG7 inhibit tumor growth in vivo
	miR-503 [219]	CyclinD1		
LOXL1-AS1	miR-let-7a-5p [220]	EGFR	Increase	Promote cell proliferation, migration, cell cycle progression; suppress apoptosis; promote tumor growth in vivo
	miR-541-3p [221]	CCND1		
PCA3	miR-1261 [79]	PRKD3	Increase	Promote proliferation, migration, invasion, xenografts growth; inhibit apoptosis and autophagy
	miR-218-5p [84]	HMGB1		
HOTAIR	miR-520b [222]	FGFR1	Increase	Promote cell proliferation, migration and invasion; reasonable biomarker for PCa bone metastasis
PVT1	miR-186/miR-146 [223,224]	Twist1	Increase	Promote EMT and cell invasion, and repress cell apoptosis
LINC00473	miR-195-5p [225]	SEPT2	Increase	Promote cell proliferation via JAK-STAT3 signaling pathway
SNHG17	miR-144 [226]	CD51	Increase	Promote cell proliferation, migration and migration
ZEB-AS1	miR-342-3p [227]	CUL4B	Increase	Promote cell proliferation, migration and invasion through PI3K/AKT/mTOR signaling pathway
LINC00665	miR-1224-5p [228]	SND1	Increase	Promote cell growth and metastasis
SNH3	miR-577 [229]	SMURF1	Increase	Promote cell proliferation, migration, EMT and resistant apoptosis
FEZF1-AS1	miR-25-3p [230]	ITGB8	Increase	Promote cell viability and EMT; Inhibit cell autophagy
CRNDE	miR-101 [231]	Rap1A	Increase	Promote cell proliferation, migration and invasion; decrease apoptosis
FER1L4	miR-92a-3p [232]	FBXW7	Decrease	Inhibit cell proliferation and promote cell apoptosis
HOXA11-AS	miR-518b [233]	ACTN4	Increase	Promote cell proliferation, migration and inhibit apoptosis
VPS9D1-AS1	miR-4739 [234]	MEF2D	Increase	Promote cell proliferation, migration, invasion and inhibit apoptosis
HCP5	miR-4656 [235]	CEMIP	Increase	Promote proliferation, colony formation and inhibit apoptosis
LSAMP-AS1	miR-183-5p [113]	DCN	Decrease	Inhibit cell proliferation migration, invasion and EMT
RBMS3-AS3	miR-4534 [236]	VASH1	Decrease	Inhibit cell proliferation, migration, invasion and angiogenesis
SNHG4	miR-377 [237]	ZIC5	Increase	Promote cell growth, migration and invasion
SHNG20	miR-6516 [238]	SCGB2A1	Increase	Promote cell proliferation, invasion and suppress apoptosis
FOXP4-AS1	miR-3184-5p [239]	FOXP4	Increase	Promote cell proliferation and decrease cell apoptosis
SNHG15	miR-338-3p [240]	FKBP1A	Increase	Promote cell proliferation, migration, invasion, viability and EMT
LEF1-AS1	miR-330 [241]	LEF1	Increase	Promote cell proliferation, invasion and migration
MEG3	miR-9-5p [97]	QKI-5	Decrease	Inhibit proliferation, migration, invasion and induce apoptosis
FOXC2-AS1	miR-1253 [242]	EZH2	Increase	Promote cell proliferation and tumor growth
MYU	miR-184 [243]	Myc	Increase	Promote cell growth and migration
PCSEAT	miR-143-3p/24-2-5p [244]	EZH2	Increase	Promote cell growth and mobility
PCAT3/PCAT9	miR-203 [245]	SNAI2	Increase	Promote cell proliferation, invasion, migration, angiogenesis and stemness
FENDRR	miR-18a-5p [208]	RUNX1	Decrease	Inhibit cell proliferation, migration, invasion and induce apoptosis
CASC2	miR-183 [114]	Sprouty2	Decrease	Inhibit cell proliferation and induce apoptosis
ANRIL	let-7a [164]	TGF-b1/Smad	Increase	Promote cell proliferation and migration
XIST	miR-23a [109]	RKIP	Decrease	Inhibit cell proliferation and tumor metastasis
GAS5	miR-103 [107]	AKT-mTOR	Decrease	Inhibit cell proliferation, invasion and migration
OGFRP1	miR-124-3p [246]	SARM1	Increase	Promote cell growth and metastasis
PROX1-AS1	miR-647 [247]		Increase	Promote cell proliferation and invasion
ZFAS1	miR-135-5p [248]		Increase	Promote proliferation, migration, invasion, EMT and inhibit apoptosis
TTN-AS1	miR-1271 [249]		Increase	Promote cell proliferation and migration
AFAP1-AS1	miR-512-3p [250]		Increase	Promote cell proliferation, migration and invasion
CASC15	miR-200a-3p [251]		Increase	Promote cell migration and invasion
HCG11	miR-543 [252]	PI3K/AKT	Decrease	Inhibit cell proliferation, invasion, migration and induce apoptosis
LINC00662	miR-34a [253]		Increase	Promote cell proliferation, invasion, migration and suppress apoptosis
LncRNA APP	miR-218 [85]	ZEB2/CDH2	Increase	Promote cell migration and invasion
CHRF	miR-10b [254]	cyclinD1/CDK4/6	Increase	Promote cell proliferation, EMT and repress apoptosis
TTTY15	let-7 [204]	CDK6/FN1	Increase	Promote cell growth in vivo and in vitro
Linc00581	miR-216b-5p [255]	GATA6	Increase	Promote cell viability
RNCR3	miR-85-5p [256]	BRD8 ISO2	Increase	Promote cell proliferation, invasion and colony formation
HOTTIP	miR-216a-5p [257]		Increase	Promote cell proliferation, migration and invasion
PCGEM1	miR-148a [258]		Increase	Inhibit early cell apoptosis
PCAT-1	miR-145-5p [259]	FSCN1	Increase	Promote proliferation, migration, invasion and repress apoptosis
lncRNA625	miR-432 [260]	TRIM29/PYGO2	Decrease	Inhibit cell proliferation and promote cell apoptosis
SChLAP1	miR-198 [53]	MAPK1 signaling	Increase	Promote cell proliferation, invasion and repress apoptosis
H19	miR-657 [261]	TGFB1		Repress cell migration and cancer metastasis

## References

[1] Miller K.D., Nogueira L., Mariotto A.B., Rowland J.H., Yabroff K.R., Alfano C.M., Jemal A., Kramer J.L., Siegel R.L. (2019). Cancer treatment and survivorship statistics, 2019. CA Cancer J. Clin..

[2] Bray F., Ferlay J., Soerjomataram I., Siegel R.L., Torre L.A., Jemal A. (2018). Global cancer statistics 2018: GLOBOCAN estimates of incidence and mortality worldwide for 36 cancers in 185 countries. CA Cancer J. Clin..

[3] Evans A.J. (2018). Treatment effects in prostate cancer. Modern pathology: An official journal of the United States and Canadian Academy of Pathology, Inc. Mod. Pathol..

[4] Gillessen S., Attard G., Beer T.M., Beltran H., Bjartell A., Bossi A., Briganti A., Bristow R.G., Chi K.N., Clarke N. (2020). Management of Patients with Advanced Prostate Cancer: Report of the Advanced Prostate Cancer Consensus Conference 2019. Eur. Urol..

[5] Altavilla A., Casadei C., Lolli C., Menna C., Ravaglia G., Gurioli G., Farolfi A., Brighi N., Conteduca V., Burgio S.L. (2020). Enzalutamide for the treatment of nonmetastatic castration-resistant prostate cancer. Expert Opin. Pharmacother..

[6] Davis I.D., Martin A.J., Stockler M.R., Begbie S., Chi K.N., Chowdhury S., Coskinas X., Frydenberg M., Hague W.E., Horvath L.G. (2019). Enzalutamide with Standard First-Line Therapy in Metastatic Prostate Cancer. N. Engl. J. Med..

[7] Fizazi K., Tran N., Fein L., Matsubara N., Rodriguez-Antolin A., Alekseev B.Y., Ozguroglu M., Ye D., Feyerabend S., Protheroe A. (2019). Abiraterone acetate plus prednisone in patients with newly diagnosed high-risk metastatic castration-sensitive prostate cancer (LATITUDE): Final overall survival analysis of a randomised, double-blind, phase 3 trial. Lancet Oncol..

[8] Fizazi K., Tran N., Fein L., Matsubara N., Rodriguez-Antolin A., Alekseev B.Y., Ozguroglu M., Ye D., Feyerabend S., Protheroe A. (2017). Abiraterone plus Prednisone in Metastatic, Castration-Sensitive Prostate Cancer. N. Engl. J. Med..

[9] Armstrong A.J., Szmulewitz R.Z., Petrylak D.P., Holzbeierlein J., Villers A., Azad A., Alcaraz A., Alekseev B., Iguchi T., Shore N.D. (2019). ARCHES: A Randomized, Phase III Study of Androgen Deprivation Therapy with Enzalutamide or Placebo in Men with Metastatic Hormone-Sensitive Prostate Cancer. J. Clin. Oncol..

[10] Finan C., Gaulton A., Kruger F.A., Lumbers R.T., Shah T., Engmann J., Galver L., Kelley R., Karlsson A., Santos R. (2017). The druggable genome and support for target identification and validation in drug development. Sci. Transl. Med..

[11] Lee R.C., Feinbaum R.L., Ambros V. (1993). The *C. elegans* heterochronic gene lin-4 encodes small RNAs with antisense complementarity to lin-14. Cell.

[12] Brannan C.I., Dees E.C., Ingram R.S., Tilghman S.M. (1990). The product of the H19 gene may function as an RNA. Mol. Cell. Biol..

[13] Nair L., Chung H., Basu U. (2020). Regulation of long non-coding RNAs and genome dynamics by the RNA surveillance machinery. Nat. Rev. Mol. Cell Biol..

[14] Thapar R. (2018). Regulation of DNA Double-Strand Break Repair by Non-Coding RNAs. Molecules.

[15] Su M., Wang H., Wang W., Wang Y., Ouyang L., Pan C., Xia L., Cao D., Liao Q. (2018). LncRNAs in DNA damage response and repair in cancer cells. Acta Biochim. Biophys. Sin..

[16] He M., Zhou W., Li C., Guo M. (2016). MicroRNAs, DNA Damage Response, and Cancer Treatment. Int. J. Mol. Sci..

[17] Denli A.M., Tops B.B., Plasterk R.H., Ketting R.F., Hannon G.J. (2004). Processing of primary microRNAs by the Microprocessor complex. Nature.

[18] O’Brien J., Hayder H., Zayed Y., Peng C. (2018). Overview of MicroRNA Biogenesis, Mechanisms of Actions, and Circulation. Front. Endocrinol..

[19] Xu W., San Lucas A., Wang Z., Liu Y. (2014). Identifying microRNA targets in different gene regions. BMC Bioinform..

[20] Bartel D.P. (2009). MicroRNAs: Target recognition and regulatory functions. Cell.

[21] Hartford C.C.R., Lal A. (2020). When Long Noncoding Becomes Protein Coding. Mol. Cell. Biol..

[22] Djebali S., Davis C.A., Merkel A., Dobin A., Lassmann T., Mortazavi A., Tanzer A., Lagarde J., Lin W., Schlesinger F. (2012). Landscape of transcription in human cells. Nature.

[23] Guttman M., Amit I., Garber M., French C., Lin M.F., Feldser D., Huarte M., Zuk O., Carey B.W., Cassady J.P. (2009). Chromatin signature reveals over a thousand highly conserved large non-coding RNAs in mammals. Nature.

[24] Dahariya S., Paddibhatla I., Kumar S., Raghuwanshi S., Pallepati A., Gutti R.K. (2019). Long non-coding RNA: Classification, biogenesis and functions in blood cells. Mol. Immunol..

[25] Yousefi H., Maheronnaghsh M., Molaei F., Mashouri L., Reza Aref A., Momeny M., Alahari S.K. (2020). Long noncoding RNAs and exosomal lncRNAs: Classification, and mechanisms in breast cancer metastasis and drug resistance. Oncogene.

[26] Ulitsky I., Bartel D.P. (2013). lincRNAs: Genomics, evolution, and mechanisms. Cell.

[27] Chen L.L. (2016). Linking Long Noncoding RNA Localization and Function. Trends Biochem. Sci..

[28] Derrien T., Johnson R., Bussotti G., Tanzer A., Djebali S., Tilgner H., Guernec G., Martin D., Merkel A., Knowles D.G. (2012). The GENCODE v7 catalog of human long noncoding RNAs: Analysis of their gene structure, evolution, and expression. Genome Res..

[29] Carlevaro-Fita J., Johnson R. (2019). Global Positioning System: Understanding Long Noncoding RNAs through Subcellular Localization. Mol. Cell.

[30] Mercer T.R., Mattick J.S. (2013). Structure and function of long noncoding RNAs in epigenetic regulation. Nat. Struct. Mol. Biol..

[31] Zhang A., Zhao J.C., Kim J., Fong K.W., Yang Y.A., Chakravarti D., Mo Y.Y., Yu J. (2015). LncRNA HOTAIR Enhances the Androgen-Receptor-Mediated Transcriptional Program and Drives Castration-Resistant Prostate Cancer. Cell Rep..

[32] Prensner J.R., Iyer M.K., Sahu A., Asangani I.A., Cao Q., Patel L., Vergara I.A., Davicioni E., Erho N., Ghadessi M. (2013). The long noncoding RNA SChLAP1 promotes aggressive prostate cancer and antagonizes the SWI/SNF complex. Nat. Genet..

[33] Gu P., Chen X., Xie R., Han J., Xie W., Wang B., Dong W., Chen C., Yang M., Jiang J. (2017). lncRNA HOXD-AS1 Regulates Proliferation and Chemo-Resistance of Castration-Resistant Prostate Cancer via Recruiting WDR5. Mol. Ther. J. Am. Soc. Gene Ther..

[34] Munschauer M., Nguyen C.T., Sirokman K., Hartigan C.R., Hogstrom L., Engreitz J.M., Ulirsch J.C., Fulco C.P., Subramanian V., Chen J. (2018). The NORAD lncRNA assembles a topoisomerase complex critical for genome stability. Nature.

[35] Wilusz J.E., Sunwoo H., Spector D.L. (2009). Long noncoding RNAs: Functional surprises from the RNA world. Genes Dev..

[36] Sen R., Ghosal S., Das S., Balti S., Chakrabarti J. (2014). Competing endogenous RNA: The key to posttranscriptional regulation. Sci. World J..

[37] Thomson D.W., Dinger M.E. (2016). Endogenous microRNA sponges: Evidence and controversy. Nat. Reviews. Genet..

[38] Lopez-Urrutia E., Montes L.P.B., Cervantes D.L.d.G., Perez-Plasencia C., Campos-Parra A.D. (2019). Crosstalk Between Long Non-coding RNAs, Micro-RNAs and mRNAs: Deciphering Molecular Mechanisms of Master Regulators in Cancer. Front. Oncol..

[39] Guttman M., Rinn J.L. (2012). Modular regulatory principles of large non-coding RNAs. Nature.

[40] Adams B.D., Anastasiadou E., Esteller M., He L., Slack F.J. (2015). The Inescapable Influence of Noncoding RNAs in Cancer. Cancer Res..

[41] Kwok Z.H., Tay Y. (2017). Long noncoding RNAs: Lincs between human health and disease. Biochem. Soc. Trans..

[42] Anastasiadou E., Jacob L.S., Slack F.J. (2018). Non-coding RNA networks in cancer. Nature reviews. Cancer.

[43] Guil S., Esteller M. (2015). RNA-RNA interactions in gene regulation: The coding and noncoding players. Trends Biochem. Sci..

[44] Salmena L., Poliseno L., Tay Y., Kats L., Pandolfi P.P. (2011). A ceRNA hypothesis: The Rosetta Stone of a hidden RNA language?. Cell.

[45] Tay Y., Rinn J., Pandolfi P.P. (2014). The multilayered complexity of ceRNA crosstalk and competition. Nature.

[46] Ebert M.S., Sharp P.A. (2010). Emerging roles for natural microRNA sponges. Curr. Biol. CB.

[47] Seitz H. (2009). Redefining microRNA targets. Curr. Biol. CB.

[48] Poliseno L., Salmena L., Zhang J., Carver B., Haveman W.J., Pandolfi P.P. (2010). A coding-independent function of gene and pseudogene mRNAs regulates tumour biology. Nature.

[49] Ebert M.S., Neilson J.R., Sharp P.A. (2007). MicroRNA sponges: Competitive inhibitors of small RNAs in mammalian cells. Nat. Methods.

[50] Jin Y., Cui Z., Li X., Jin X., Peng J. (2017). Upregulation of long non-coding RNA PlncRNA-1 promotes proliferation and induces epithelial-mesenchymal transition in prostate cancer. Oncotarget.

[51] Cui Z., Ren S., Lu J., Wang F., Xu W., Sun Y., Wei M., Chen J., Gao X., Xu C. (2013). The prostate cancer-up-regulated long noncoding RNA PlncRNA-1 modulates apoptosis and proliferation through reciprocal regulation of androgen receptor. Urol. Oncol..

[52] Fang Z., Xu C., Li Y., Cai X., Ren S., Liu H., Wang Y., Wang F., Chen R., Qu M. (2016). A feed-forward regulatory loop between androgen receptor and PlncRNA-1 promotes prostate cancer progression. Cancer Lett..

[53] Li Y., Luo H., Xiao N., Duan J., Wang Z., Wang S. (2018). Long Noncoding RNA SChLAP1 Accelerates the Proliferation and Metastasis of Prostate Cancer via Targeting miR-198 and Promoting the MAPK1 Pathway. Oncol. Res..

[54] Gutschner T., Hammerle M., Eissmann M., Hsu J., Kim Y., Hung G., Revenko A., Arun G., Stentrup M., Gross M. (2013). The noncoding RNA MALAT1 is a critical regulator of the metastasis phenotype of lung cancer cells. Cancer Res..

[55] Xu S., Sui S., Zhang J., Bai N., Shi Q., Zhang G., Gao S., You Z., Zhan C., Liu F. (2015). Downregulation of long noncoding RNA MALAT1 induces epithelial-to-mesenchymal transition via the PI3K-AKT pathway in breast cancer. Int. J. Clin. Exp. Pathol..

[56] Ren S., Liu Y., Xu W., Sun Y., Lu J., Wang F., Wei M., Shen J., Hou J., Gao X. (2013). Long noncoding RNA MALAT-1 is a new potential therapeutic target for castration resistant prostate cancer. J. Urol..

[57] Chang J., Xu W., Du X., Hou J. (2018). MALAT1 silencing suppresses prostate cancer progression by upregulating miR-1 and downregulating KRAS. Onco. Targets Ther..

[58] Dai X., Liang Z., Liu L., Guo K., Xu S., Wang H. (2019). Silencing of MALAT1 inhibits migration and invasion by sponging miR13p in prostate cancer cells. Mol. Med. Rep..

[59] Dai X., Liu L., Liang Z., Guo K., Xu S., Wang H. (2019). Silencing of lncRNA MALAT1 inhibits cell cycle progression via androgen receptor signaling in prostate cancer cells. Pathol. Res. Pract..

[60] Xue D., Lu H., Xu H.Y., Zhou C.X., He X.Z. (2018). Long noncoding RNA MALAT1 enhances the docetaxel resistance of prostate cancer cells via miR-145-5p-mediated regulation of AKAP12. J. Cell Mol. Med..

[61] Wang F., Ren S., Chen R., Lu J., Shi X., Zhu Y., Zhang W., Jing T., Zhang C., Shen J. (2014). Development and prospective multicenter evaluation of the long noncoding RNA MALAT-1 as a diagnostic urinary biomarker for prostate cancer. Oncotarget.

[62] Hamilton M.P., Rajapakshe K.I., Bader D.A., Cerne J.Z., Smith E.A., Coarfa C., Hartig S.M., McGuire S.E. (2016). The Landscape of microRNA Targeting in Prostate Cancer Defined by AGO-PAR-CLIP. Neoplasia.

[63] Jiang X., Guo S., Zhang Y., Zhao Y., Li X., Jia Y., Xu Y., Ma B. (2020). LncRNA NEAT1 promotes docetaxel resistance in prostate cancer by regulating ACSL4 via sponging miR-34a-5p and miR-204-5p. Cell Signal.

[64] Guo Z., He C., Yang F., Qin L., Lu X., Wu J. (2019). Long non-coding RNA-NEAT1, a sponge for miR-98-5p, promotes expression of oncogene HMGA2 in prostate cancer. Biosci. Rep..

[65] Wang F., Li X., Xie X., Zhao L., Chen W. (2008). UCA1, a non-protein-coding RNA up-regulated in bladder carcinoma and embryo, influencing cell growth and promoting invasion. FEBS Lett..

[66] Zhang S., Dong X., Ji T., Chen G., Shan L. (2017). Long non-coding RNA UCA1 promotes cell progression by acting as a competing endogenous RNA of ATF2 in prostate cancer. Am. J. Transl. Res..

[67] Ghafouri-Fard S., Taheri M. (2019). UCA1 long non-coding RNA: An update on its roles in malignant behavior of cancers. Biomed. Pharmacother..

[68] Wang X., Yang B., Ma B. (2016). The UCA1/miR-204/Sirt1 axis modulates docetaxel sensitivity of prostate cancer cells. Cancer Chemother Pharmacol..

[69] He C., Lu X., Yang F., Qin L., Guo Z., Sun Y., Wu J. (2019). LncRNA UCA1 acts as a sponge of miR-204 to up-regulate CXCR4 expression and promote prostate cancer progression. Biosci. Rep..

[70] Yu Y., Gao F., He Q., Li G., Ding G. (2020). lncRNA UCA1 Functions as a ceRNA to Promote Prostate Cancer Progression via Sponging miR143. Mol. Ther. Nucleic Acids.

[71] Zhou Y., Wang X., Zhang J., He A., Wang Y.L., Han K., Su Y., Yin J., Lv X., Hu H. (2017). Artesunate suppresses the viability and mobility of prostate cancer cells through UCA1, the sponge of miR-184. Oncotarget.

[72] Young T.L., Matsuda T., Cepko C.L. (2005). The noncoding RNA taurine upregulated gene 1 is required for differentiation of the murine retina. Curr. Biol. CB.

[73] Ghaforui-Fard S., Vafaee R., Taheri M. (2019). Taurine-upregulated gene 1: A functional long noncoding RNA in tumorigenesis. J. Cell. Physiol..

[74] Yang X.L., Wei C., Zhang Y.B., Guo H.Q. (2019). Long noncoding RNA TUG1 promotes progression via upregulating DGCR8 in prostate cancer. Eur. Rev. Med. Pharmacol. Sci..

[75] Xiu D., Liu L., Cheng M., Sun X., Ma X. (2020). Knockdown of lncRNA TUG1 Enhances Radiosensitivity of Prostate Cancer via the TUG1/miR-139-5p/SMC1A Axis. Onco. Targets Ther..

[76] Li G., Yang J., Chong T., Huang Y., Liu Y., Li H. (2020). TUG1 knockdown inhibits the tumorigenesis and progression of prostate cancer by regulating microRNA-496/Wnt/beta-catenin pathway. Anticancer. Drugs.

[77] Yang B., Tang X., Wang Z., Sun D., Wei X., Ding Y. (2018). TUG1 promotes prostate cancer progression by acting as a ceRNA of miR-26a. Biosci. Rep..

[78] Lemos A.E.G., Matos A.D.R., Ferreira L.B., Gimba E.R.P. (2019). The long non-coding RNA PCA3: An update of its functions and clinical applications as a biomarker in prostate cancer. Oncotarget.

[79] He J.H., Li B.X., Han Z.P., Zou M.X., Wang L., Lv Y.B., Zhou J.B., Cao M.R., Li Y.G., Zhang J.Z. (2016). Snail-activated long non-coding RNA PCA3 up-regulates PRKD3 expression by miR-1261 sponging, thereby promotes invasion and migration of prostate cancer cells. Tumour. Biol..

[80] Peng P., Chen T., Wang Q., Zhang Y., Zheng F., Huang S., Tang Y., Yang C., Ding W., Ren D. (2019). Decreased miR-218-5p Levels as a Serum Biomarker in Bone Metastasis of Prostate Cancer. Oncol. Res. Treat..

[81] Tian J., Zhang H., Mu L., Wang M., Li X., Zhang X., Xie E., Ma M., Wu D., Du Y. (2020). The miR-218/GAB2 axis regulates proliferation, invasion and EMT via the PI3K/AKT/GSK-3beta pathway in prostate cancer. Exp. Cell Res..

[82] Guan B., Wu K., Zeng J., Xu S., Mu L., Gao Y., Wang K., Ma Z., Tian J., Shi Q. (2017). Tumor-suppressive microRNA-218 inhibits tumor angiogenesis via targeting the mTOR component RICTOR in prostate cancer. Oncotarget.

[83] Li F., Gu C., Tian F., Jia Z., Meng Z., Ding Y., Yang J. (2016). MiR-218 impedes IL-6-induced prostate cancer cell proliferation and invasion via suppression of LGR4 expression. Oncol. Rep..

[84] Zhang G., He X., Ren C., Lin J., Wang Q. (2019). Long noncoding RNA PCA3 regulates prostate cancer through sponging miR-218-5p and modulating high mobility group box 1. J. Cell. Physiol..

[85] Shi X., Zhang W., Nian X., Lu X., Li Y., Liu F., Wang F., He B., Zhao L., Zhu Y. (2020). The previously uncharacterized lncRNA APP promotes prostate cancer progression by acting as a competing endogenous RNA. Int. J. Cancer.

[86] Wang X., He C., Yang Z., Li S., Qiao L., Fang L. (2019). Dysregulation of long non-coding RNA SNHG12 alters the viability, apoptosis, and autophagy of prostate cancer cells by regulating miR-195/CCNE1 axis. Int. J. Clin. Exp. Pathol..

[87] Song J., Wu X., Ma R., Miao L., Xiong L., Zhao W. (2019). Long noncoding RNA SNHG12 promotes cell proliferation and activates Wnt/beta-catenin signaling in prostate cancer through sponging microRNA-195. J. Cell Biochem..

[88] Cheng G., Song Z., Liu Y., Xiao H., Ruan H., Cao Q., Wang K., Xiao W., Xiong Z., Liu D. (2020). Long noncoding RNA SNHG12 indicates the prognosis of prostate cancer and accelerates tumorigenesis via sponging miR-133b. J. Cell. Physiol..

[89] Schaid D.J., McDonnell S.K., FitzGerald L.M., DeRycke L., Fogarty Z., Giles G.G., MacInnis R.J., Southey M.C., Nguyen-Dumont T., Cancel-Tassin G. (2021). Two-stage Study of Familial Prostate Cancer by Whole-exome Sequencing and Custom Capture Identifies 10 Novel Genes Associated with the Risk of Prostate Cancer. Eur. Urol..

[90] Prensner J.R., Chen W., Han S., Iyer M.K., Cao Q., Kothari V., Evans J.R., Knudsen K.E., Paulsen M.T., Ljungman M. (2014). The long non-coding RNA PCAT-1 promotes prostate cancer cell proliferation through cMyc. Neoplasia.

[91] Walavalkar K., Saravanan B., Singh A.K., Jayani R.S., Nair A., Farooq U., Islam Z., Soota D., Mann R., Shivaprasad P.V. (2020). A rare variant of African ancestry activates 8q24 lncRNA hub by modulating cancer associated enhancer. Nat. Commun..

[92] Guo H., Wu Y., Nouri M., Spisak S., Russo J.W., Sowalsky A.G., Pomerantz M.M., Wei Z., Korthauer K., Seo J.H. (2021). Androgen receptor and MYC equilibration centralizes on developmental super-enhancer. Nat. Commun..

[93] Slack F.J., Chinnaiyan A.M. (2019). The Role of Non-coding RNAs in Oncology. Cell.

[94] Ghafouri-Fard S., Taheri M. (2019). Maternally expressed gene 3 (MEG3): A tumor suppressor long non coding RNA. Biomed. Pharmacother..

[95] Zhou Y., Zhong Y., Wang Y., Zhang X., Batista D.L., Gejman R., Ansell P.J., Zhao J., Weng C., Klibanski A. (2007). Activation of p53 by MEG3 non-coding RNA. J. Biol. Chem..

[96] Moradi M.T., Fallahi H., Rahimi Z. (2019). Interaction of long noncoding RNA MEG3 with miRNAs: A reciprocal regulation. J. Cell Biochem..

[97] Wu M., Huang Y., Chen T., Wang W., Yang S., Ye Z., Xi X. (2019). LncRNA MEG3 inhibits the progression of prostate cancer by modulating miR-9-5p/QKI-5 axis. J. Cell Mol. Med..

[98] Luo G., Wang M., Wu X., Tao D., Xiao X., Wang L., Min F., Zeng F., Jiang G. (2015). Long Non-Coding RNA MEG3 Inhibits Cell Proliferation and Induces Apoptosis in Prostate Cancer. Cell Physiol. Biochem..

[99] Goustin A.S., Thepsuwan P., Kosir M.A., Lipovich L. (2019). The Growth-Arrest-Specific (GAS)-5 Long Non-Coding RNA: A Fascinating lncRNA Widely Expressed in Cancers. Noncoding RNA.

[100] Nam R.K., Zhang W.W., Loblaw D.A., Klotz L.H., Trachtenberg J., Jewett M.A., Stanimirovic A., Davies T.O., Toi A., Venkateswaran V. (2008). A genome-wide association screen identifies regions on chromosomes 1q25 and 7p21 as risk loci for sporadic prostate cancer. Prostate Cancer Prostatic Dis..

[101] Mourtada-Maarabouni M., Hasan A.M., Farzaneh F., Williams G.T. (2010). Inhibition of human T-cell proliferation by mammalian target of rapamycin (mTOR) antagonists requires noncoding RNA growth-arrest-specific transcript 5 (GAS5). Mol. Pharmacol..

[102] Smith C.M., Steitz J.A. (1998). Classification of gas5 as a multi-small-nucleolar-RNA (snoRNA) host gene and a member of the 5’-terminal oligopyrimidine gene family reveals common features of snoRNA host genes. Mol. Cell. Biol..

[103] Tani H., Torimura M., Akimitsu N. (2013). The RNA degradation pathway regulates the function of GAS5 a non-coding RNA in mammalian cells. PLoS ONE.

[104] Mourtada-Maarabouni M., Williams G.T. (2013). Growth arrest on inhibition of nonsense-mediated decay is mediated by noncoding RNA GAS5. Biomed. Res. Int..

[105] Isken O., Maquat L.E. (2007). Quality control of eukaryotic mRNA: Safeguarding cells from abnormal mRNA function. Genes Dev..

[106] Yacqub-Usman K., Pickard M.R., Williams G.T. (2015). Reciprocal regulation of GAS5 lncRNA levels and mTOR inhibitor action in prostate cancer cells. Prostate.

[107] Xue D., Zhou C., Lu H., Xu R., Xu X., He X. (2016). LncRNA GAS5 inhibits proliferation and progression of prostate cancer by targeting miR-103 through AKT/mTOR signaling pathway. Tumour. Biol..

[108] Yang Z., Jiang X., Jiang X., Zhao H. (2018). X-inactive-specific transcript: A long noncoding RNA with complex roles in human cancers. Gene.

[109] Du Y., Weng X.D., Wang L., Liu X.H., Zhu H.C., Guo J., Ning J.Z., Xiao C.C. (2017). LncRNA XIST acts as a tumor suppressor in prostate cancer through sponging miR-23a to modulate RKIP expression. Oncotarget.

[110] Abdelmohsen K., Panda A., Kang M.J., Xu J., Selimyan R., Yoon J.H., Martindale J.L., De S., Wood W.H., Becker K.G. (2013). Senescence-associated lncRNAs: Senescence-associated long noncoding RNAs. Aging Cell.

[111] Petrovics G., Li H., Stumpel T., Tan S.H., Young D., Katta S., Li Q., Ying K., Klocke B., Ravindranath L. (2015). A novel genomic alteration of LSAMP associates with aggressive prostate cancer in African American men. EBioMedicine.

[112] Goyal A., Fiskin E., Gutschner T., Polycarpou-Schwarz M., Gross M., Neugebauer J., Gandhi M., Caudron-Herger M., Benes V., Diederichs S. (2017). A cautionary tale of sense-antisense gene pairs: Independent regulation despite inverse correlation of expression. Nucleic Acids Res..

[113] Hua X., Liu Z., Zhou M., Tian Y., Zhao P.P., Pan W.H., Li C.X., Huang X.X., Liao Z.X., Xian Q. (2019). LSAMP-AS1 binds to microRNA-183-5p to suppress the progression of prostate cancer by up-regulating the tumor suppressor DCN. EBioMedicine.

[114] Gao W., Lin S., Cheng C., Zhu A., Hu Y., Shi Z., Zhang X., Hong Z. (2019). Long non-coding RNA CASC2 regulates Sprouty2 via functioning as a competing endogenous RNA for miR-183 to modulate the sensitivity of prostate cancer cells to docetaxel. Arch. Biochem. Biophys..

[115] McKie A.B., Douglas D.A., Olijslagers S., Graham J., Omar M.M., Heer R., Gnanapragasam V.J., Robson C.N., Leung H.Y. (2005). Epigenetic inactivation of the human sprouty2 (hSPRY2) homologue in prostate cancer. Oncogene.

[116] Mihelich B.L., Khramtsova E.A., Arva N., Vaishnav A., Johnson D.N., Giangreco A.A., Martens-Uzunova E., Bagasra O., Kajdacsy-Balla A., Nonn L. (2011). miR-183-96-182 cluster is overexpressed in prostate tissue and regulates zinc homeostasis in prostate cells. J. Biol. Chem..

[117] Larne O., Ostling P., Haflidadottir B.S., Hagman Z., Aakula A., Kohonen P., Kallioniemi O., Edsjo A., Bjartell A., Lilja H. (2015). miR-183 in prostate cancer cells positively regulates synthesis and serum levels of prostate-specific antigen. Eur. Urol..

[118] Katz B., Reis S.T., Viana N.I., Morais D.R., Moura C.M., Dip N., Silva I.A., Iscaife A., Srougi M., Leite K.R. (2014). Comprehensive study of gene and microRNA expression related to epithelial-mesenchymal transition in prostate cancer. PLoS ONE.

[119] Ueno K., Hirata H., Shahryari V., Deng G., Tanaka Y., Tabatabai Z.L., Hinoda Y., Dahiya R. (2013). microRNA-183 is an oncogene targeting Dkk-3 and SMAD4 in prostate cancer. Br. J. Cancer.

[120] Waseem M., Ahmad M.K., Serajuddin M., Bhaskar V., Sankhwar S.N., Mahdi A.A. (2019). MicroRNA-183-5p: A New Potential Marker for Prostate Cancer. Indian J. Clin. Biochem..

[121] Li Y., He S., Zhan Y., He A., Gong Y., Ji G., Huang C., Peng D., Guan B., Li X. (2019). microRNA-183-3p Inhibits Progression of Human Prostate Cancer by Downregulating High-Mobility Group Nucleosome Binding Domain 5. DNA Cell Biol..

[122] Lindahl T., Barnes D.E. (2000). Repair of endogenous DNA damage. Cold Spring Harbor Symposia on Quantitative Biology.

[123] Yasui A., Kanno S., Takao M. (2003). DNA damage, repair and aging. Nihon Ronen Igakkai Zasshi.

[124] Branzei D., Foiani M. (2008). Regulation of DNA repair throughout the cell cycle. Nat. Rev. Mol. Cell Biol..

[125] Krokan H.E., Bjoras M. (2013). Base excision repair. Cold Spring Harb. Perspect. Biol..

[126] Kobayashi T., Takeuchi S., Saijo M., Nakatsu Y., Morioka H., Otsuka E., Wakasugi M., Nikaido O., Tanaka K. (1998). Mutational analysis of a function of xeroderma pigmentosum group A (XPA) protein in strand-specific DNA repair. Nucleic Acids Res..

[127] Sugasawa K., Ng J.M., Masutani C., Iwai S., van der Spek P.J., Eker A.P., Hanaoka F., Bootsma D., Hoeijmakers J.H. (1998). Xeroderma pigmentosum group C protein complex is the initiator of global genome nucleotide excision repair. Mol. Cell.

[128] Ganai R.A., Johansson E. (2016). DNA Replication-A Matter of Fidelity. Mol. Cell.

[129] Edelbrock M.A., Kaliyaperumal S., Williams K.J. (2009). DNA mismatch repair efficiency and fidelity are elevated during DNA synthesis in human cells. Mutat. Res..

[130] Wei W., Ba Z., Gao M., Wu Y., Ma Y., Amiard S., White C.I., Rendtlew Danielsen J.M., Yang Y.G., Qi Y. (2012). A role for small RNAs in DNA double-strand break repair. Cell.

[131] Gao M., Wei W., Li M.M., Wu Y.S., Ba Z., Jin K.X., Li M.M., Liao Y.Q., Adhikari S., Chong Z. (2014). Ago2 facilitates Rad51 recruitment and DNA double-strand break repair by homologous recombination. Cell Res..

[132] Price B.D., D’Andrea A.D. (2013). Chromatin remodeling at DNA double-strand breaks. Cell.

[133] Michelini F., Pitchiaya S., Vitelli V., Sharma S., Gioia U., Pessina F., Cabrini M., Wang Y., Capozzo I., Iannelli F. (2017). Damage-induced lncRNAs control the DNA damage response through interaction with DDRNAs at individual double-strand breaks. Nat. Cell Biol..

[134] Wang Q., Goldstein M. (2016). Small RNAs Recruit Chromatin-Modifying Enzymes MMSET and Tip60 to Reconfigure Damaged DNA upon Double-Strand Break and Facilitate Repair. Cancer Res..

[135] Burger K., Schlackow M., Potts M., Hester S., Mohammed S., Gullerova M. (2017). Nuclear phosphorylated Dicer processes double-stranded RNA in response to DNA damage. J. Cell Biol..

[136] Cabrini M., Roncador M., Galbiati A., Cipolla L., Maffia A., Iannelli F., Sabbioneda S., d’Adda di Fagagna F., Francia S. (2021). DROSHA is recruited to DNA damage sites by the MRN complex to promote non-homologous end joining. J. Cell Sci..

[137] Francia S., Michelini F., Saxena A., Tang D., de Hoon M., Anelli V., Mione M., Carninci P., d’Adda di Fagagna F. (2012). Site-specific DICER and DROSHA RNA products control the DNA-damage response. Nature.

[138] Bonath F., Domingo-Prim J., Tarbier M., Friedlander M.R., Visa N. (2018). Next-generation sequencing reveals two populations of damage-induced small RNAs at endogenous DNA double-strand breaks. Nucleic Acids Res..

[139] Lee S., Kopp F., Chang T.C., Sataluri A., Chen B., Sivakumar S., Yu H., Xie Y., Mendell J.T. (2016). Noncoding RNA NORAD Regulates Genomic Stability by Sequestering PUMILIO Proteins. Cell.

[140] Tichon A., Perry R.B., Stojic L., Ulitsky I. (2018). SAM68 is required for regulation of Pumilio by the NORAD long noncoding RNA. Genes Dev..

[141] Elguindy M.M., Kopp F., Goodarzi M., Rehfeld F., Thomas A., Chang T.C., Mendell J.T. (2019). PUMILIO, but not RBMX, binding is required for regulation of genomic stability by noncoding RNA NORAD. eLife.

[142] Tichon A., Gil N., Lubelsky Y., Havkin Solomon T., Lemze D., Itzkovitz S., Stern-Ginossar N., Ulitsky I. (2016). A conserved abundant cytoplasmic long noncoding RNA modulates repression by Pumilio proteins in human cells. Nat. Commun..

[143] Miao Z., Guo X., Tian L. (2019). The long noncoding RNA NORAD promotes the growth of gastric cancer cells by sponging miR-608. Gene.

[144] Li Q., Li C., Chen J., Liu P., Cui Y., Zhou X., Li H., Zu X. (2018). High expression of long noncoding RNA NORAD indicates a poor prognosis and promotes clinical progression and metastasis in bladder cancer. Urol. Oncol..

[145] Wu X., Lim Z.F., Li Z., Gu L., Ma W., Zhou Q., Su H., Wang X., Yang X., Zhang Z. (2017). NORAD Expression Is Associated with Adverse Prognosis in Esophageal Squamous Cell Carcinoma. Oncol. Res. Treat.

[146] Wang L., Du L., Duan W., Yan S., Xie Y., Wang C. (2018). Overexpression of long noncoding RNA NORAD in colorectal cancer associates with tumor progression. Onco Targets Ther..

[147] He H., Yang H., Liu D., Pei R. (2019). LncRNA NORAD promotes thyroid carcinoma progression through targeting miR-202-5p. Am. J. Transl. Res..

[148] Gao W., Weng T., Wang L., Shi B., Meng W., Wang X., Wu Y., Jin L., Fei L. (2019). Long noncoding RNA NORAD promotes cell proliferation and glycolysis in nonsmall cell lung cancer by acting as a sponge for miR1365p. Mol. Med. Rep..

[149] Yang Z., Zhao Y., Lin G., Zhou X., Jiang X., Zhao H. (2019). Noncoding RNA activated by DNA damage (NORAD): Biologic function and mechanisms in human cancers. Clin. Chim. Acta.

[150] Zhang H., Guo H. (2019). Long non-coding RNA NORAD induces cell proliferation and migration in prostate cancer. J. Int. Med. Res..

[151] Choi E.H., Kim K.P. (2019). E2F1 facilitates DNA break repair by localizing to break sites and enhancing the expression of homologous recombination factors. Exp. Mol. Med..

[152] Roy R., Chun J., Powell S.N. (2011). BRCA1 and BRCA2: Different roles in a common pathway of genome protection. Nat. Rev. Cancer.

[153] Prensner J.R., Chen W., Iyer M.K., Cao Q., Ma T., Han S., Sahu A., Malik R., Wilder-Romans K., Navone N. (2014). PCAT-1, a long noncoding RNA, regulates BRCA2 and controls homologous recombination in cancer. Cancer Res..

[154] Shen L., Wang Q., Liu R., Chen Z., Zhang X., Zhou P., Wang Z. (2018). LncRNA lnc-RI regulates homologous recombination repair of DNA double-strand breaks by stabilizing RAD51 mRNA as a competitive endogenous RNA. Nucleic Acids Res..

[155] Liu Y., Xu X., Xu X., Li S., Liang Z., Hu Z., Wu J., Zhu Y., Jin X., Wang X. (2017). MicroRNA-193a-3p inhibits cell proliferation in prostate cancer by targeting cyclin D1. Oncol. Lett..

[156] Jirawatnotai S., Hu Y., Michowski W., Elias J.E., Becks L., Bienvenu F., Zagozdzon A., Goswami T., Wang Y.E., Clark A.B. (2011). A function for cyclin D1 in DNA repair uncovered by protein interactome analyses in human cancers. Nature.

[157] Barr A.R., Cooper S., Heldt F.S., Butera F., Stoy H., Mansfeld J., Novak B., Bakal C. (2017). DNA damage during S-phase mediates the proliferation-quiescence decision in the subsequent G1 via p21 expression. Nat. Commun..

[158] Zhang N., Kaur R., Akhter S., Legerski R.J. (2009). Cdc5L interacts with ATR and is required for the S-phase cell-cycle checkpoint. EMBO Rep..

[159] Bernstein H.S., Coughlin S.R. (1998). A mammalian homolog of fission yeast Cdc5 regulates G2 progression and mitotic entry. J. Biol. Chem..

[160] Li X., Wang X., Song W., Xu H., Huang R., Wang Y., Zhao W., Xiao Z., Yang X. (2018). Oncogenic Properties of NEAT1 in Prostate Cancer Cells Depend on the CDC5L-AGRN Transcriptional Regulation Circuit. Cancer Res..

[161] Adriaens C., Standaert L., Barra J., Latil M., Verfaillie A., Kalev P., Boeckx B., Wijnhoven P.W., Radaelli E., Vermi W. (2016). p53 induces formation of NEAT1 lncRNA-containing paraspeckles that modulate replication stress response and chemosensitivity. Nat. Med..

[162] Wan G., Mathur R., Hu X., Liu Y., Zhang X., Peng G., Lu X. (2013). Long non-coding RNA ANRIL (CDKN2B-AS) is induced by the ATM-E2F1 signaling pathway. Cell Signal.

[163] Yap K.L., Li S., Munoz-Cabello A.M., Raguz S., Zeng L., Mujtaba S., Gil J., Walsh M.J., Zhou M.M. (2010). Molecular interplay of the noncoding RNA ANRIL and methylated histone H3 lysine 27 by polycomb CBX7 in transcriptional silencing of INK4a. Mol. Cell.

[164] Zhao B., Lu Y.L., Yang Y., Hu L.B., Bai Y., Li R.Q., Zhang G.Y., Li J., Bi C.W., Yang L.B. (2018). Overexpression of lncRNA ANRIL promoted the proliferation and migration of prostate cancer cells via regulating let-7a/TGF-beta1/ Smad signaling pathway. Cancer Biomark..

[165] Miao J.T., Gao J.H., Chen Y.Q., Chen H., Meng H.Y., Lou G. (2019). LncRNA ANRIL affects the sensitivity of ovarian cancer to cisplatin via regulation of let-7a/HMGA2 axis. Biosci. Rep..

[166] Wang Y., Cheng N., Luo J. (2017). Downregulation of lncRNA ANRIL represses tumorigenicity and enhances cisplatin-induced cytotoxicity via regulating microRNA let-7a in nasopharyngeal carcinoma. J. Biochem. Mol. Toxicol..

[167] Sharma V., Khurana S., Kubben N., Abdelmohsen K., Oberdoerffer P., Gorospe M., Misteli T. (2015). A BRCA1-interacting lncRNA regulates homologous recombination. EMBO Rep..

[168] Stiff T., O’Driscoll M., Rief N., Iwabuchi K., Lobrich M., Jeggo P.A. (2004). ATM and DNA-PK function redundantly to phosphorylate H2AX after exposure to ionizing radiation. Cancer Res..

[169] Gottlieb T.M., Jackson S.P. (1993). The DNA-dependent protein kinase: Requirement for DNA ends and association with Ku antigen. Cell.

[170] Bjorkman A., Du L., Felgentreff K., Rosner C., Pankaj Kamdar R., Kokaraki G., Matsumoto Y., Davies E.G., van der Burg M., Notarangelo L.D. (2015). DNA-PKcs Is Involved in Ig Class Switch Recombination in Human B Cells. J. Immunol..

[171] Shrivastav M., De Haro L.P., Nickoloff J.A. (2008). Regulation of DNA double-strand break repair pathway choice. Cell Res..

[172] Tamang S., Acharya V., Roy D., Sharma R., Aryaa A., Sharma U., Khandelwal A., Prakash H., Vasquez K.M., Jain A. (2019). SNHG12: An LncRNA as a Potential Therapeutic Target and Biomarker for Human Cancer. Front. Oncol..

[173] Haemmig S., Yang D., Sun X., Das D., Ghaffari S., Molinaro R., Chen L., Deng Y., Freeman D., Moullan N. (2020). Long noncoding RNA SNHG12 integrates a DNA-PK-mediated DNA damage response and vascular senescence. Sci. Transl. Med..

[174] Cai C., Chen Q.B., Han Z.D., Zhang Y.Q., He H.C., Chen J.H., Chen Y.R., Yang S.B., Wu Y.D., Zeng Y.R. (2015). miR-195 Inhibits Tumor Progression by Targeting RPS6KB1 in Human Prostate Cancer. Clin. Cancer Res..

[175] Rafiei S., Fitzpatrick K., Liu D., Cai M.Y., Elmarakeby H.A., Park J., Ricker C., Kochupurakkal B.S., Choudhury A.D., Hahn W.C. (2020). ATM Loss Confers Greater Sensitivity to ATR Inhibition Than PARP Inhibition in Prostate Cancer. Cancer Res..

[176] Diaz-Mejia N., Garcia-Illescas D., Morales-Barrera R., Suarez C., Planas J., Maldonado X., Carles J., Mateo J. (2021). PARP inhibitors in advanced prostate cancer: When to use them?. Endocr. Relat. Cancer.

[177] Mateo J., Porta N., Bianchini D., McGovern U., Elliott T., Jones R., Syndikus I., Ralph C., Jain S., Varughese M. (2020). Olaparib in patients with metastatic castration-resistant prostate cancer with DNA repair gene aberrations (TOPARP-B): A multicentre, open-label, randomised, phase 2 trial. Lancet Oncol..

[178] Quigley D., Alumkal J.J., Wyatt A.W., Kothari V., Foye A., Lloyd P., Aggarwal R., Kim W., Lu E., Schwartzman J. (2017). Analysis of Circulating Cell-Free DNA Identifies Multiclonal Heterogeneity of BRCA2 Reversion Mutations Associated with Resistance to PARP Inhibitors. Cancer Discov..

[179] Dhuri K., Bechtold C., Quijano E., Pham H., Gupta A., Vikram A., Bahal R. (2020). Antisense Oligonucleotides: An Emerging Area in Drug Discovery and Development. J. Clin. Med..

[180] Maruyama R., Yokota T. (2020). Knocking Down Long Noncoding RNAs Using Antisense Oligonucleotide Gapmers. Methods Mol. Biol..

[181] Wahlestedt C., Salmi P., Good L., Kela J., Johnsson T., Hokfelt T., Broberger C., Porreca F., Lai J., Ren K. (2000). Potent and nontoxic antisense oligonucleotides containing locked nucleic acids. Proc. Natl. Acad. Sci. USA.

[182] Mangos M.M., Min K.L., Viazovkina E., Galarneau A., Elzagheid M.I., Parniak M.A., Damha M.J. (2003). Efficient RNase H-directed cleavage of RNA promoted by antisense DNA or 2’F-ANA constructs containing acyclic nucleotide inserts. J. Am. Chem. Soc..

[183] Lennox K.A., Behlke M.A. (2016). Cellular localization of long non-coding RNAs affects silencing by RNAi more than by antisense oligonucleotides. Nucleic Acids Res..

[184] Crooke S.T., Wang S., Vickers T.A., Shen W., Liang X.H. (2017). Cellular uptake and trafficking of antisense oligonucleotides. Nat Biotechnol..

[185] Food & Drug Administration FDA Approves First-of-Its Kind Targeted RNA-Based Therapy to Treat a Rare Disease. https://www.fda.gov/news-events/press-announcements/fda-approves-first-its-kind-targeted-rna-based-therapy-treat-rare-diseaseF.a.f.-o.-i.k.t.R.-b.t.t.t.a.r.d.A.

[186] Wurster C.D., Winter B., Wollinsky K., Ludolph A.C., Uzelac Z., Witzel S., Schocke M., Schneider R., Kocak T. (2019). Intrathecal administration of nusinersen in adolescent and adult SMA type 2 and 3 patients. J. Neurol..

[187] Bajan S., Hutvagner G. (2020). RNA-Based Therapeutics: From Antisense Oligonucleotides to miRNAs. Cells.

[188] Bonneau E., Neveu B., Kostantin E., Tsongalis G.J., De Guire V. (2019). How close are miRNAs from clinical practice? A perspective on the diagnostic and therapeutic market. EJIFCC.

[189] https://clinicaltrials.gov/ct2/results?term=lncRNA.

[190] Fletcher C.E., Sulpice E., Combe S., Shibakawa A., Leach D.A., Hamilton M.P., Chrysostomou S.L., Sharp A., Welti J., Yuan W. (2019). Androgen receptor-modulatory microRNAs provide insight into therapy resistance and therapeutic targets in advanced prostate cancer. Oncogene.

[191] Chowdhury S., Burris H.A., Patel M., Infante J.R., Jones S.F., Voskoboynik M., Parry K., Elvin P., Coleman T., Arkenau H.T. A phase I dose escalation, safety and pharmacokinetic (PK) study of AZD5312 (IONIS-ARRx), a first-in-class Generation 2.5 antisense oligonucleotide targeting the androgen receptor (AR). Proceedings of the European Journal of Cancer. Conference: 28th EORTC-NCI-AACR Symposium on Molecular Targets and Cancer Therapeutics.

[192] Hatano K., Kumar B., Zhang Y., Coulter J.B., Hedayati M., Mears B., Ni X., Kudrolli T.A., Chowdhury W.H., Rodriguez R. (2015). A functional screen identifies miRNAs that inhibit DNA repair and sensitize prostate cancer cells to ionizing radiation. Nucleic Acids Res..

[193] Mao A., Zhao Q., Zhou X., Sun C., Si J., Zhou R., Gan L., Zhang H. (2016). MicroRNA-449a enhances radiosensitivity by downregulation of c-Myc in prostate cancer cells. Sci. Rep..

[194] Tao Z., Xu S., Ruan H., Wang T., Song W., Qian L., Chen K. (2018). MiR-195/-16 Family Enhances Radiotherapy via T Cell Activation in the Tumor Microenvironment by Blocking the PD-L1 Immune Checkpoint. Cell Physiol. Biochem..

[195] Neijenhuis S., Bajrami I., Miller R., Lord C.J., Ashworth A. (2013). Identification of miRNA modulators to PARP inhibitor response. DNA Repair.

[196] Zhang X., Jin K., Luo J.D., Liu B., Xie L.P. (2019). MicroRNA-107 inhibits proliferation of prostate cancer cells by targeting cyclin E1. Neoplasma.

[197] Mercatelli N., Coppola V., Bonci D., Miele F., Costantini A., Guadagnoli M., Bonanno E., Muto G., Frajese G.V., De Maria R. (2008). The inhibition of the highly expressed miR-221 and miR-222 impairs the growth of prostate carcinoma xenografts in mice. PLoS ONE.

[198] Berton S., Cusan M., Segatto I., Citron F., D’Andrea S., Benevol S., Avanzo M., Dall’Acqua A., Schiappacassi M., Bristow R.G. (2017). Loss of p27(kip1) increases genomic instability and induces radio-resistance in luminal breast cancer cells. Sci. Rep..

[199] Ariel I., Lustig O., Schneider T., Pizov G., Sappir M., De-Groot N., Hochberg A. (1995). The imprinted H19 gene as a tumor marker in bladder carcinoma. Urology.

[200] Ariel I., Sughayer M., Fellig Y., Pizov G., Ayesh S., Podeh D., Libdeh B.A., Levy C., Birman T., Tykocinski M.L. (2000). The imprinted H19 gene is a marker of early recurrence in human bladder carcinoma. Mol. Pathol..

[201] Zhang Y., Pitchiaya S., Cieslik M., Niknafs Y.S., Tien J.C., Hosono Y., Iyer M.K., Yazdani S., Subramaniam S., Shukla S.K. (2018). Analysis of the androgen receptor-regulated lncRNA landscape identifies a role for ARLNC1 in prostate cancer progression. Nat. Genet..

[202] Zhang W., Shi X., Chen R., Zhu Y., Peng S., Chang Y., Nian X., Xiao G., Fang Z., Li Y. (2020). Novel Long Non-coding RNA lncAMPC Promotes Metastasis and Immunosuppression in Prostate Cancer by Stimulating LIF/LIFR Expression. Mol. Ther. J. Am. Soc. Gene Ther..

[203] Dowdy S.F. (2017). Overcoming cellular barriers for RNA therapeutics. Nat. Biotechnol..

[204] Xiao G., Yao J., Kong D., Ye C., Chen R., Li L., Zeng T., Wang L., Zhang W., Shi X. (2019). The Long Noncoding RNA TTTY15, Which Is Located on the Y Chromosome, Promotes Prostate Cancer Progression by Sponging let-7. Eur. Urol..

[205] Guo B., Wu S., Zhu X., Zhang L., Deng J., Li F., Wang Y., Zhang S., Wu R., Lu J. (2020). Micropeptide CIP2A-BP encoded by LINC00665 inhibits triple-negative breast cancer progression. EMBO J..

[206] Joung J., Konermann S., Gootenberg J.S., Abudayyeh O.O., Platt R.J., Brigham M.D., Sanjana N.E., Zhang F. (2017). Genome-scale CRISPR-Cas9 knockout and transcriptional activation screening. Nat. Protoc..

[207] Lee C.S., Bishop E.S., Zhang R., Yu X., Farina E.M., Yan S., Zhao C., Zheng Z., Shu Y., Wu X. (2017). Adenovirus-Mediated Gene Delivery: Potential Applications for Gene and Cell-Based Therapies in the New Era of Personalized Medicine. Genes Dis..

[208] Zhang G., Han G., Zhang X., Yu Q., Li Z., Li Z., Li J. (2018). Long non-coding RNA FENDRR reduces prostate cancer malignancy by competitively binding miR-18a-5p with RUNX1. Biomarkers.

[209] Adams D., Gonzalez-Duarte A., O’Riordan W.D., Yang C.C., Ueda M., Kristen A.V., Tournev I., Schmidt H.H., Coelho T., Berk J.L. (2018). Patisiran, an RNAi Therapeutic, for Hereditary Transthyretin Amyloidosis. N. Engl. J. Med..

[210] Anand P., Stahel V.P. (2021). Review the safety of Covid-19 mRNA vaccines: A review. Patient Saf. Surg..

[211] Luo Z.F., Peng Y., Liu F.H., Ma J.S., Hu G., Lai S.L., Lin H., Chen J.J., Zou G.M., Yan Q. (2020). Long noncoding RNA SNHG14 promotes malignancy of prostate cancer by regulating with miR-5590-3p/YY1 axis. Eur. Rev. Med. Pharmacol. Sci..

[212] Song X., Wang H., Wu J., Sun Y. (2020). Long Noncoding RNA SOX2-OT Knockdown Inhibits Proliferation and Metastasis of Prostate Cancer Cells Through Modulating miR-452-5p/HMGB3 Axis and Inactivating Wnt/beta-Catenin Pathway. Cancer Biother. Radiopharm..

[213] Wo Q., Zhang D., Hu L., Lyu J., Xiang F., Zheng W., Shou J., Qi X. (2019). Long noncoding RNA SOX2-OT facilitates prostate cancer cell proliferation and migration via miR-369-3p/CFL2 axis. Biochem. Biophys. Res. Commun..

[214] Xie M., Zhang Z., Cui Y. (2020). Long Noncoding RNA SNHG1 Contributes to the Promotion of Prostate Cancer Cells Through Regulating miR-377-3p/AKT2 Axis. Cancer Biother. Radiopharm..

[215] Li J., Zhang Z., Xiong L., Guo C., Jiang T., Zeng L., Li G., Wang J. (2017). SNHG1 lncRNA negatively regulates miR-199a-3p to enhance CDK7 expression and promote cell proliferation in prostate cancer. Biochem. Biophys. Res. Commun..

[216] Ma Y., Fan B., Ren Z., Liu B., Wang Y. (2019). Long noncoding RNA DANCR contributes to docetaxel resistance in prostate cancer through targeting the miR-34a-5p/JAG1 pathway. Onco Targets Ther..

[217] Zhao H.F., Zhang Z.C., Shi B.K., Jiang X.Z. (2019). DANCR sponges miR-135a to regulate paclitaxel sensitivity in prostate cancer. Eur. Rev. Med. Pharmacol. Sci..

[218] Han Y., Hu H., Zhou J. (2019). Knockdown of LncRNA SNHG7 inhibited epithelial-mesenchymal transition in prostate cancer though miR-324-3p/WNT2B axis in vitro. Pathol. Res. Pract..

[219] Qi H., Wen B., Wu Q., Cheng W., Lou J., Wei J., Huang J., Yao X., Weng G. (2018). Long noncoding RNA SNHG7 accelerates prostate cancer proliferation and cycle progression through cyclin D1 by sponging miR-503. Biomed. Pharmacother..

[220] Bai T., Liu Y., Li B. (2019). LncRNA LOXL1-AS1/miR-let-7a-5p/EGFR-related pathway regulates the doxorubicin resistance of prostate cancer DU-145 cells. IUBMB Life.

[221] Long B., Li N., Xu X.X., Li X.X., Xu X.J., Liu J.Y., Wu Z.H. (2018). Long noncoding RNA LOXL1-AS1 regulates prostate cancer cell proliferation and cell cycle progression through miR-541-3p and CCND1. Biochem. Biophys. Res. Commun..

[222] Zhang J.J., Zhou X.H., Zhou Y., Wang Y.G., Qian B.Z., He A.N., Shen Z., Hu H.Y., Yao Y. (2019). Bufalin suppresses the migration and invasion of prostate cancer cells through HOTAIR, the sponge of miR-520b. Acta Pharmacol. Sin..

[223] Chang Z., Cui J., Song Y. (2018). Long noncoding RNA PVT1 promotes EMT via mediating microRNA-186 targeting of Twist1 in prostate cancer. Gene.

[224] Liu H.T., Fang L., Cheng Y.X., Sun Q. (2016). LncRNA PVT1 regulates prostate cancer cell growth by inducing the methylation of miR-146a. Cancer Med..

[225] Xing Z., Li S., Liu Z., Zhang C., Meng M., Bai Z. (2020). The long non-coding RNA LINC00473 contributes to cell proliferation via JAK-STAT3 signaling pathway by regulating miR-195-5p/SEPT2 axis in prostate cancer. Biosci. Rep..

[226] Bai M., Lei Y., Wang M., Ma J., Yang P., Mou X., Dong Y., Han S. (2020). Long Non-coding RNA SNHG17 Promotes Cell Proliferation and Invasion in Castration-Resistant Prostate Cancer by Targeting the miR-144/CD51 Axis. Front. Genet..

[227] Ma T., Chen H., Wang P., Yang N., Bao J. (2020). Downregulation of lncRNA ZEB1-AS1 Represses Cell Proliferation, Migration, and Invasion Through Mediating PI3K/AKT/mTOR Signaling by miR-342-3p/CUL4B Axis in Prostate Cancer. Cancer Biother. Radiopharm..

[228] Chen W., Yu Z., Huang W., Yang Y., Wang F., Huang H. (2020). LncRNA LINC00665 Promotes Prostate Cancer Progression via miR-1224-5p/SND1 Axis. Onco Targets Ther..

[229] Li T., Xing Y., Yang F., Sun Y., Zhang S., Wang Q., Zhang W. (2020). LncRNA SNHG3 sponges miR-577 to up-regulate SMURF1 expression in prostate cancer. Cancer Med..

[230] Wang Z.H., Wang J.H., Wang K.Q., Zhou Y., Wang J. (2020). LncRNA FEZF1-AS1 promoted chemoresistance, autophagy and epithelial-mesenchymal transition (EMT) through regulation of miR-25-3p/ITGB8 axis in prostate cancer. Eur. Rev. Med. Pharmacol. Sci..

[231] Chen J.H., Tong W., Pu X.F., Wang J.Z. (2020). Long noncoding RNA CRNDE promotes proliferation, migration and invasion in prostate cancer through miR-101/Rap1A. Neoplasma.

[232] Huo W., Qi F., Wang K. (2020). Long non-coding RNA FER1L4 inhibits prostate cancer progression via sponging miR-92a-3p and upregulation of FBXW7. Cancer Cell. Int..

[233] Xing Z., Li S., Liu Z., Zhang C., Bai Z. (2020). CTCF-induced upregulation of HOXA11-AS facilitates cell proliferation and migration by targeting miR-518b/ACTN4 axis in prostate cancer. Prostate.

[234] Wang X., Chen Q., Wang X., Li W., Yu G., Zhu Z., Zhang W. (2020). ZEB1 activated-VPS9D1-AS1 promotes the tumorigenesis and progression of prostate cancer by sponging miR-4739 to upregulate MEF2D. Biomed. Pharmacother..

[235] Hu R., Lu Z. (2020). Long noncoding RNA HCP5 promotes prostate cancer cell proliferation by acting as the sponge of miR4656 to modulate CEMIP expression. Oncol. Rep..

[236] Jiang Z., Zhang Y., Chen X., Wu P., Chen D. (2020). Long noncoding RNA RBMS3-AS3 acts as a microRNA-4534 sponge to inhibit the progression of prostate cancer by upregulating VASH1. Gene Ther..

[237] Wang Z.Y., Duan Y., Wang P. (2020). SP1-mediated upregulation of lncRNA SNHG4 functions as a ceRNA for miR-377 to facilitate prostate cancer progression through regulation of ZIC5. J. Cell Physiol..

[238] Wu X., Xiao Y., Zhou Y., Zhou Z., Yan W. (2019). lncRNA SNHG20 promotes prostate cancer migration and invasion via targeting the miR-6516-5p/SCGB2A1 axis. Am. J. Transl. Res..

[239] Wu X., Xiao Y., Zhou Y., Zhou Z., Yan W. (2019). LncRNA FOXP4-AS1 is activated by PAX5 and promotes the growth of prostate cancer by sequestering miR-3184-5p to upregulate FOXP4. Cell Death Dis..

[240] Zhang Y., Zhang D., Lv J., Wang S., Zhang Q. (2019). LncRNA SNHG15 acts as an oncogene in prostate cancer by regulating miR-338-3p/FKBP1A axis. Gene.

[241] Liu D.C., Song L.L., Liang Q., Hao L., Zhang Z.G., Han C.H. (2019). Long noncoding RNA LEF1-AS1 silencing suppresses the initiation and development of prostate cancer by acting as a molecular sponge of miR-330-5p via LEF1 repression. J. Cell Physiol..

[242] Chen Y., Gu M., Liu C., Wan X., Shi Q., Chen Q., Wang Z. (2019). Long noncoding RNA FOXC2-AS1 facilitates the proliferation and progression of prostate cancer via targeting miR-1253/EZH2. Gene.

[243] Wang J., Yang X., Li R., Wang L., Gu Y., Zhao Y., Huang K.H., Cheng T., Yuan Y., Gao S. (2018). Long non-coding RNA MYU promotes prostate cancer proliferation by mediating the miR-184/c-Myc axis. Oncol. Rep..

[244] Yang X., Wang L., Li R., Zhao Y., Gu Y., Liu S., Cheng T., Huang K., Yuan Y., Song D. (2018). The long non-coding RNA PCSEAT exhibits an oncogenic property in prostate cancer and functions as a competing endogenous RNA that associates with EZH2. Biochem. Biophys. Res Commun..

[245] Tao F., Tian X., Zhang Z. (2018). The PCAT3/PCAT9-miR-203-SNAI2 axis functions as a key mediator for prostate tumor growth and progression. Oncotarget.

[246] Yan K., Hou L., Liu T., Jiao W., Ma Q., Fang Z., Zhang S., Song D., Liu J., Gao X. (2020). lncRNA OGFRP1 functions as a ceRNA to promote the progression of prostate cancer by regulating SARM1 level via miR-124-3p. Aging.

[247] Qian C., Liao C.H., Tan B.F., Chen Y.F., Dang B.W., Chen J.L., Liu C.B. (2020). LncRNA PROX1-AS1 promotes proliferation, invasion, and migration in prostate cancer via targeting miR-647. Eur. Rev. Med. Pharmacol. Sci..

[248] Pan J., Xu X., Wang G. (2020). lncRNA ZFAS1 Is Involved in the Proliferation, Invasion and Metastasis of Prostate Cancer Cells Through Competitively Binding to miR-135a-5p. Cancer Manag. Res..

[249] Zhu Y., Yang Z., Luo X.H., Xu P. (2019). Long noncoding RNA TTN-AS1 promotes the proliferation and migration of prostate cancer by inhibiting miR-1271 level. Eur. Rev. Med. Pharmacol. Sci..

[250] Wang K., Sun H., Sun T., Qu H., Xie Q., Lv H., Hu B. (2020). Long non-coding RNA AFAP1-AS1 promotes proliferation and invasion in prostate cancer via targeting miR-512-3p. Gene.

[251] Zhang C., Wang G.X., Fu B., Zhou X.C., Li Y., Li Y.Y. (2019). LncRNA CASC15 promotes migration and invasion in prostate cancer via targeting miR-200a-3p. Eur. Rev. Med. Pharmacol. Sci..

[252] Wang Y.C., He W.Y., Dong C.H., Pei L., Ma Y.L. (2019). lncRNA HCG11 regulates cell progression by targeting miR-543 and regulating AKT/mTOR pathway in prostate cancer. Cell Biol. Int..

[253] Li N., Zhang L.Y., Qiao Y.H., Song R.J. (2019). Long noncoding RNA LINC00662 functions as miRNA sponge to promote the prostate cancer tumorigenesis through targeting miR-34a. Eur. Rev. Med. Pharmacol. Sci..

[254] Liu S., Wang L., Li Y., Cui Y., Wang Y., Liu C. (2019). Long non-coding RNA CHRF promotes proliferation and mesenchymal transition (EMT) in prostate cancer cell line PC3 requiring up-regulating microRNA-10b. Biol. Chem..

[255] He J., Sun M., Geng H., Tian S. (2019). Long non-coding RNA Linc00518 promotes paclitaxel resistance of the human prostate cancer by sequestering miR-216b-5p. Biol. Cell.

[256] Tian C., Deng Y., Jin Y., Shi S., Bi H. (2018). Long non-coding RNA RNCR3 promotes prostate cancer progression through targeting miR-185-5p. Am. J. Transl. Res.

[257] Yang B., Gao G., Wang Z., Sun D., Wei X., Ma Y., Ding Y. (2018). Long non-coding RNA HOTTIP promotes prostate cancer cells proliferation and migration by sponging miR-216a-5p. Biosci. Rep..

[258] Zhang S., Li Z., Zhang L., Xu Z. (2018). MEF2activated long noncoding RNA PCGEM1 promotes cell proliferation in hormonerefractory prostate cancer through downregulation of miR148a. Mol. Med. Rep..

[259] Xu W., Chang J., Du X., Hou J. (2017). Long non-coding RNA PCAT-1 contributes to tumorigenesis by regulating FSCN1 via miR-145-5p in prostate cancer. Biomed. Pharmacother..

[260] Li J.B., Liu F., Zhang B.P., Bai W.K., Cheng W., Zhang Y.H., Yu L.J. (2017). LncRNA625 modulates prostate cancer cells proliferation and apoptosis through regulating the Wnt/beta-catenin pathway by targeting miR-432. Eur. Rev. Med. Pharmacol. Sci..

[261] Zhu M., Chen Q., Liu X., Sun Q., Zhao X., Deng R., Wang Y., Huang J., Xu M., Yan J. (2014). lncRNA H19/miR-675 axis represses prostate cancer metastasis by targeting TGFBI. FEBS J..

